# Hypoxia, metastatic origin and HPV16 E6/E7 expression differentially shape the radiation response in head and neck squamous cell carcinoma cell lines

**DOI:** 10.1038/s41598-026-54319-0

**Published:** 2026-05-22

**Authors:** Jana Pereckova, Filip Zavadil Kokas, Simona Voznicova, Ondrej Vasicek, Jitka Holcakova, Roman Hrstka, Tomas Perecko

**Affiliations:** 1 Department of Cell Biology and Radiobiology, Institute of Biophysics of the Czech Academy of Sciences, Kralovopolska 135, 612 00 Brno, Czech Republic; 2 Research Centre for Applied Molecular Oncology, Masaryk Memorial Cancer Institute, Zluty kopec 7, 656 53 Brno, Czech Republic; 3 Department of Biophysics of Immune System, Institute of Biophysics of the Czech Academy of Sciences, Kralovopolska 135, 612 00 Brno, Czech Republic

**Keywords:** Cell cycle, Cytokines, E6/E7 oncogene expression, Head and neck squamous cell carcinoma, Hypoxia, Ionizing radiation, Cancer, Cell biology, Molecular biology, Oncology

## Abstract

Hypoxia, a common feature of head and neck squamous cell carcinoma (HNSCC), significantly compromises radiotherapy efficacy by modulating DNA repair, cell‑cycle progression, and inflammatory signaling pathways. In addition, intrinsic tumor heterogeneity—including tumor origin and human papillomavirus (HPV) oncogene expression—further shapes therapeutic response. Despite these recognized factors, the integrated impact of oxygen tension, tumor origin, and viral oncogene expression on radiation response remains poorly defined. We examined the effects of hypoxia (1% O_2_) and gamma-irradiation (6 Gy) on proliferation, cell-cycle progression, apoptosis, transcriptomic profiles, and cytokine secretion in three HNSCC cell lines: FaDu (primary origin), Detroit-562 (metastatic origin), and 2A3, an isogenic FaDu derivative stably expressing HPV-16 E6/E7 oncogenes. Hypoxia prolonged cell doubling time across all lines, independent of origin or E6/E7 expression, while irradiation induced features of an intermediate epithelial–mesenchymal transition phenotype. Compared with FaDu, 2A3 cells exhibited distinct transcriptional responses, reduced cytokine secretion (IL-8, Serpin E1), and altered cell-cycle regulation following irradiation. Detroit-562 cells secreted markedly higher cytokine levels and showed no increase in cleaved caspase-3 under hypoxia after irradiation. Our findings demonstrate that oxygen tension and biological factors—including metastatic origin and E6/E7 expression—modulate radiation response in HNSCC cell lines. These results highlight the need for biomarker-driven strategies that consider both hypoxic tumor microenvironment and key biological factors such as metastatic origin and HPV‑related E6/E7 oncogene expression.

**Supplementary Information:**

The online version contains supplementary material available at 10.1038/s41598-026-54319-0.

## Introduction

Hypoxia within the tumor microenvironment promotes tumor development, progression, and metastasis, and is associated with multiple factors that influence the responses to anticancer treatments, including radiotherapy (reviewed in^1^). Tumor cell radiosensitivity is known to decrease significantly under hypoxic conditions^2^. Long-term exposure of cells to moderate hypoxia prior to irradiation has been shown to result in pronounced, cell line-specific differences in radiation effects, indicating distinct mechanisms of hypoxia adaptation^3^.

Head and neck squamous cell carcinoma (HNSCC) ranks among the ten most common cancers worldwide^4^, and approximately 80% of patients with HNSCC have an indication for radiotherapy^5^. HNSCC tumors are commonly characterized by hypoxia, which contributes to poor prognosis and reduced sensitivity to radiotherapy (reviewed in^6^. HNSCC can be classified by human papillomavirus (HPV) status: HPV-negative tumors are generally associated with tobacco or alcohol abuse, whereas HPV-positive tumors– accounting for more than 70% of oropharyngeal cancers–are primarily linked to HPV-16 infection^6^. The carcinogenic effects of HPV are primarily mediated by the E6 and E7 oncoproteins, which inhibit the functions of p53 and the retinoblastoma tumor suppressor protein, respectively^7^. HPV status may influence several biological characteristics of HNSCC. Notably, HPV-positive HNSCC cells exhibit a pronounced G2/M arrest following irradiation, reflecting impaired DNA double-strand break repair rather than enhanced apoptosis or a functional p53 response^8^. Although HPV-positive cells generally demonstrate increased radiosensitivity compared to HPV-negative cells, they exhibit similar relative radioresistance under hypoxic conditions^9^. This underscores the need for integrated analyses to guide personalized radiotherapy strategies.

Other components of the HNSCC tumor microenvironment, such as cytokines and chemokines, may reflect disease status, treatment outcome, or intrinsic (e.g., HPV status) and extrinsic (e.g., hypoxia) characteristics of the tumor cells^10^. Several cytokines have been reported to be elevated in patients with HNSCC—such as interleukin-8 (IL-8), vascular endothelial growth factor, and macrophage migration inhibitory factor (MIF), whereas others have been described as decreased (e.g., IL-2, IL-4, Serpin E1 (PAI-1) - hereafter Serpin E1, GROα) or unchanged (e.g., G-CSF, IFN-γ, TNFα) when compared with healthy controls. Notably, findings for certain cytokines, including IL-6, IL-8, and Serpin E1, have been inconsistent across studies^10–13^.

Despite these well-established findings, many mechanistic studies on cancer cells *in vitro* are still conducted under ambient air conditions. The objective of our study was to compare the responses of HNSCC cell lines to gamma-irradiation under ambient and hypoxic conditions, as well as between primary and metastatic origins, while considering the expression of HPV oncogenes. To this end, we analyzed three HNSCC cell lines: FaDu (primary origin), Detroit-562 (metastatic origin), and 2A3, an isogenic derivative of FaDu stably expressing HPV-16 E6/E7 oncogenes. We focused on assessing proliferation, cell cycle progression, apoptosis, and cytokine and chemokine production following radiation and hypoxia exposure. Additionally, we performed transcriptomic profiling to identify condition-specific gene expression signatures associated with hypoxia and radiation response in two isogenic HNSCC cell lines. To enhance the translational relevance of our findings, we further performed validation analyses in publicly available HNSCC tumor datasets. These findings may help refine biomarker-driven strategies for personalized radiotherapy.

## Materials and methods

### Cell culture

The human cell line FaDu (305033, Lot# 305033−101022, squamous cell carcinoma of the hypopharynx), and Detroit-562 (300399, Lot# 300399-719, metastatic cells of a pharyngeal carcinoma), were obtained from Cytion, Germany. The 2A3 cell line (CRL-3212, Lot# 70031242; ATCC, USA) is commercially available isogenic derivative of FaDu, generated by transfecting FaDu cells with HPV-16 E6 and E7 genes using the PA317 LXSN 16E6/E7 retroviral vector^14^. Cells were cultured in RPMI medium (21875-091, Gibco, USA) supplemented with 10% low-endotoxin fetal bovine serum (FBS, FB-1101/500, Biosera, France) and 1% penicillin/streptomycin (LM-A4118/100, Biosera, France). For 2A3 cells, penicillin/streptomycin was replaced with 0.2 mg/ml geneticin (10131-035, Gibco, USA). Cell lines were authenticated via STR profiling by the vendor. Mycoplasma contamination tests were negative. Cells were cultured at 37 °C in a humidified atmosphere under either normoxic (21% O_2_, 5% CO_2_) or hypoxic (1% O_2_, 5% CO_2_, 94% N_2_; InvivO_2_ 400, Baker Ruskinn, UK) conditions. All cell lines were cultured in parallel under normoxic and hypoxic conditions. Cells were maintained under their respective oxygen conditions continuously (FaDu and Detroit-562) or for seven days prior to experiments (2A3). Doubling time was calculated from non-irradiated controls cultured under ambient or hypoxic conditions.

### Conditioned medium

For conditioned medium collection, 2 × 10⁵ cells (FaDu, Detroit-562, and 2A3) were plated in duplicate in 6-well plates in 1.5 ml of RPMI medium supplemented with 10% FBS under either 21% or 1% O_2_. The following day, cells were irradiated with 0 or 6 Gy of gamma rays (dose rate: 1 Gy/min) using ⁶⁰Co gamma-ray source (Chisostat, Chirana, Czech Republic). Hypoxic samples were placed in airtight pouches during irradiation. After irradiation, the medium was replaced with fresh oxygen-matched RPMI + 10% FBS, and cells were incubated under their respective oxygen conditions for 24 or 48 h. At the designated time points, conditioned medium was aspirated (pooled from duplicates), centrifuged at 500×g for 10 min at 4 °C to remove cell debris, and transferred to new tubes. Samples were processed under the corresponding oxygen conditions, aliquoted, and stored at − 20 °C until further use.

### Cell cycle assay

Cells were seeded and treated as described above. Twenty-four hours after irradiation, they were harvested separately under normoxic or hypoxic conditions using oxygen-matched buffers and trypsin. Cell cycle distribution was assessed using the Muse Cell Cycle Kit (#MCH100106, Cytek, USA) according to the manufacturer’s protocol. Results are presented as the percentage of cells in G0/G1, S, or G2/M phases.

### Western blot

Cells were seeded and treated as shown above. Forty-eight hours after irradiation, they were harvested separately under normoxic or hypoxic conditions using oxygen-matched buffers and trypsin. The cells were processed as previously^15^. Membranes were incubated overnight at 4 °C with primary antibodies: anti-β-actin (1:1 000; #3700, Cell Signaling Technology, USA), N-cadherin (1:1 000; #610921, BD, USA), vimentin (1:1 000; #GTX636980, GeneTex, USA), E-cadherin (1:500; #GTX636577 GeneTex) or anti-cleaved caspase-3 (1:1 000; #9664, Cell Signaling Technology), anti HIF-1α (1:500; #SAB2702132R, Sigma, USA), and anti-human CA IX mouse monoclonal antibody M75 in undiluted hybridoma medium^16,17^. This was followed by incubation with corresponding HRP-linked secondary antibodies (anti-mouse IgG, #7076, 1:5 000; or anti-rabbit IgG, #7074, 1:2 000; both from Cell Signaling Technology) for 1 h at room temperature. Protein bands were visualized using an enhanced chemiluminescent substrate (SuperSignal 34577/34095, Thermo Fisher Scientific, USA). Chemiluminescent signals were acquired using an Amersham A680 Imager (GE Healthcare, USA) with incremental or manual exposure acquisition and monitoring of saturated pixels to ensure signals remained within the linear detection range or with Syngene Imaging System. Brightness adjustments were performed during image acquisition and applied uniformly to the entire membrane; no post-acquisition image processing was performed. Optical densities of detected proteins were measured using ImageJ v1.52 (NIH, USA).

### Cytokine array

Cytokine and chemokine profiling was performed using the Human Cytokine Array Kit (#ARY005B, Biotechne, R&D Systems, USA). Conditioned medium was collected from the three cell lines cultured under normoxic or hypoxic conditions 48 h after irradiation (non-irradiated controls were processed in parallel). Medium from three independent experiments was pooled for analysis. Following the manufacturer’s instructions, membranes were exposed using an Amersham A680 Imager (GE, USA) for 1–20 min. Optical densities of detected cytokines and chemokines were quantified with ImageJ. Background-subtracted data were normalized to positive controls, log₁₀-transformed, and converted to z-scores.

### ELISA

Levels of IL-8, MIF, and Serpin E1 were quantified using an enzyme-linked immunosorbent assay (ELISA) kits (DY208-05, DY289 and DY1786, respectively; R&D Systems) according to the manufacturer’s instructions. Immediately after irradiation, the culture medium was replaced with fresh, oxygen‑matched medium. Conditioned medium was collected 24 and 48 h after irradiation from cells maintained under normoxic or hypoxic conditions; non-irradiated controls were processed in parallel.

### RNA isolation, sequencing and analysis of differential expression

**Fig. 1:**
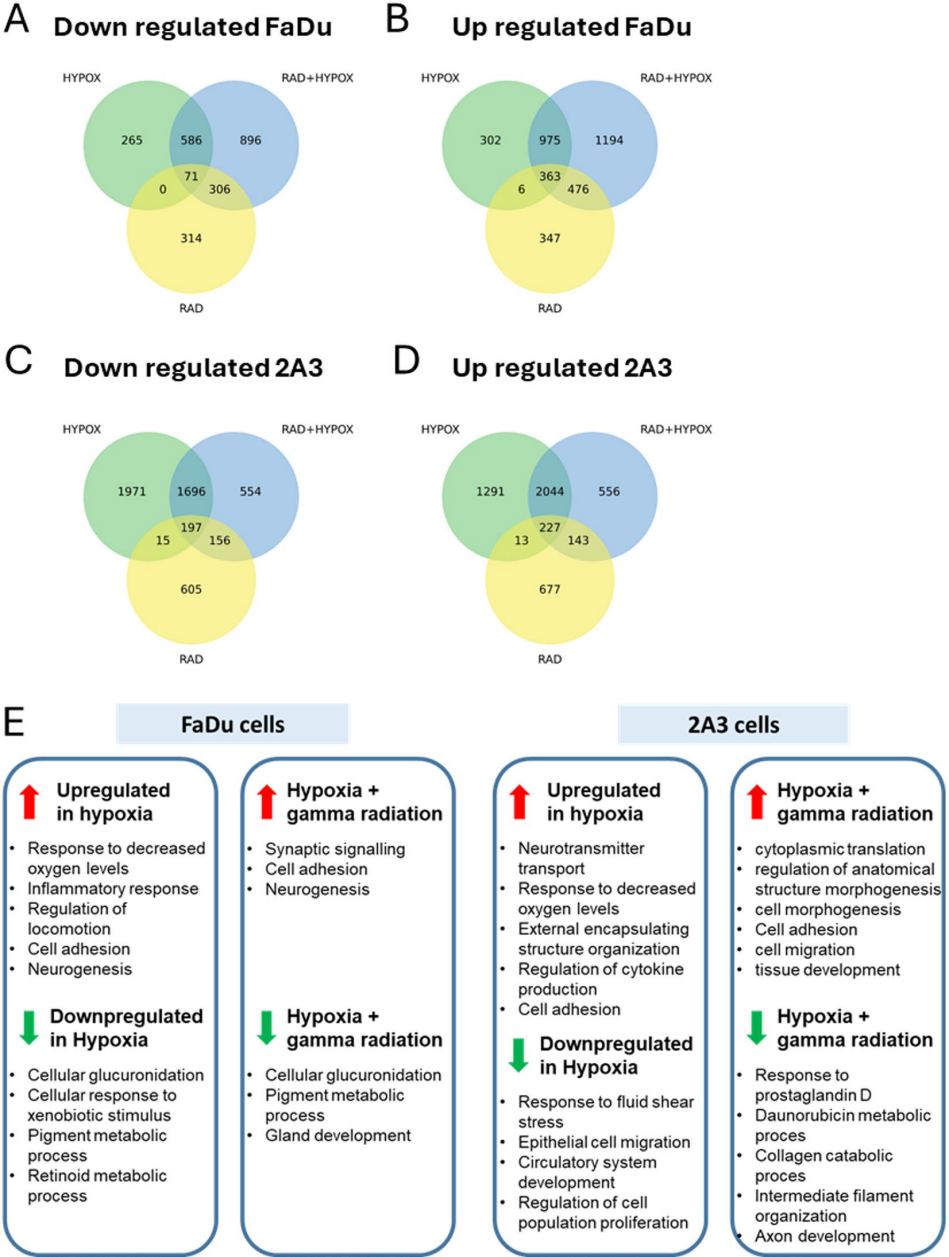
Transcriptomic responses to hypoxia and irradiation in HNSCC. (**A**–**D**) Venn diagrams of differentially expressed genes in FaDu (**A**,**B**) and 2A3 (**C**,**D**) cell lines. Venn diagrams illustrate the number of significantly regulated genes under three conditions: hypoxia (green), irradiation (yellow), and the combination of hypoxia and irradiation (blue). Numbers in the overlapping areas indicate genes commonly regulated under the corresponding conditions. Genes were considered differentially expressed if they had an adjusted p-value (padj) < 0.1. (**E**) Biological processes differentially regulated in FaDu and 2A3 HNSCC cell lines under hypoxia and combined hypoxia with gamma irradiation. The figure illustrates biological processes significantly upregulated (red) or downregulated (green) in FaDu and 2A3 cells in response to hypoxia alone or in combination with gamma radiation. The analysis was performed using the WebGestalt 2024 platform and included only genes that were consistently detected across all three independent biological replicates and met the inclusion criteria of adjusted p-value < 0.1 and |log_2_FC| ≥ 2.

Transcriptomic analyses were performed 24 h after 0 or 6 Gy irradiation under normoxic (21% O_2_) or hypoxic (1% O_2_) conditions. Total RNA was extracted from cells by TRI-Reagent (Sigma-Aldrich, USA). Only RNA samples with RNA integrity number ≥ 7 determined by a Bioanalyzer (RNA 6000 Nano Kit, Agilent Technologies, USA) passed to library preparation. The TruSeq Stranded Total RNA LT Sample Prep Kit (Illumina, USA) was used to convert 0.5 µg of total RNA into a library of template molecules. The library was validated using Bioanalyzer (DNA 1000 Kit, Agilent) and quantified according to the manufacturer’s instructions by qPCR (KAPA Library Quantification Kit Illumina platforms, Kapa Biosystems, USA) on a Quant studio 5 Real-Time PCR System (Thermo Fisher Scientific, USA). Sequencing was performed on NextSeq 500 (Illumina). Low-quality reads were removed from the raw sequencing data, adaptor sequences clipped, and low-quality leading or trailing regions (below phred score 18) were trimmed using Trimmomatic v0.33 and BBDuk2 (both from the Joint Genome Institute, USA). RNA sequencing data obtained from three biological replicates per group were aligned to the hg38^18^ reference genome using the HISAT^19^ aligner. Reads mapped to gene regions were subsequently quantified with FeatureCounts^20^, and differential expression analysis was performed using DESeq2^21^. Genes were considered differentially expressed if they had an adjusted p-value (padj) < 0.1 (in all analyses except Fig. 1E). Genes which were evaluated as differentially expressed were visualized via InteractiVenn^22^ and iDEP^23^. WEB-based GEne SeT AnaLysis Toolkit 2024 (WebGestalt 2024), using Over-Representation Analysis based on Fisher’s exact test, was employed to analyze biological processes associated with differentially expressed genes (DEGs) using curated functional databases, including Gene Ontology (GO), KEGG, and Reactome^24,25^. DEGs were identified from three independent RNA samples per cell line. DEGS that were included into the analysis had padj < 0.1 and absolute log2FC ≥ 2 (only in Fig. 1E, to decrease the number of DEGs).

### Statistics

Data are presented as arithmetic means ± standard deviations (SD). Statistical analysis was performed using two-way ANOVA followed by Tukey’s post hoc test. For comparisons between two independent groups, Student’s t-test was used. A p-value of < 0.05 was considered statistically significant. Statistical analyses were conducted using GraphPad Prism v10.4 (GraphPad Software, USA, http://www.graphpad.com). Statistical methods and the number of independent experiments are provided in the respective figure legends.

## Results

### E6/E7 expression reshapes transcriptional responses to hypoxia and irradiation in isogenic HNSCC cells

To investigate whether HPV-16 E6/E7 expression alters hypoxia‑ and irradiation‑induced stress adaptation at the transcriptional level, we analyzed DEGs and enriched biological processes in isogenic FaDu and 2A3 HNSCC cells exposed to hypoxia, irradiation, or their combination (Supplementary file 1). The total numbers of DEGs detected under each condition are summarized in Fig. 1A–D, while volcano plots illustrating the magnitude and direction of transcriptional changes are shown in Supplementary file 2, Figure S1.

To directly compare hypoxia- and irradiation-driven transcriptional programs between isogenic cell lines, we next examined enriched biological processes associated with DEGs (Fig. 1E). Under hypoxic conditions alone, FaDu and 2A3 cells exhibited distinct adaptive responses characterized by a coordinated transcriptional program aimed at enhancing survival, promoting tissue remodeling, and enabling communication within a stressed microenvironment. In both cell lines, downregulated genes were predominantly involved in biosynthetic, metabolic, and structural processes, indicating suppression of energy-demanding functions under oxygen limitation (Fig. 1E).

Upon combined exposure to hypoxia and gamma-irradiation, FaDu and 2A3 cells exhibited cell line-specific transcriptional responses (Fig. 1E). In FaDu cells, upregulated genes were enriched in pathways related to synaptic signaling, neurogenesis, and cell adhesion, potentially reflecting stress-induced plasticity and neural-like reprogramming, a phenomenon observed in epithelial tumors following genotoxic stress and often associated with treatment resistance. In contrast, 2A3 cells responded with a broad activation of morphogenetic and structural processes, including cytoplasmic translation, morphogenesis, migration, and neurogenesis suggesting a reparative or pro-survival shift possibly linked to radio-adaptive plasticity.

Analysis of downregulated pathways further highlighted these differences. In FaDu cells, suppressed genes were associated with glucuronidation, pigment metabolism, and gland development, pointing to further suppression of differentiation and metabolic stability under compounded stress. Downregulated genes in 2A3 were associated with daunorubicin metabolism, collagen catabolism, intermediate filament organization, and axon development, indicating suppression of matrix remodeling and certain structural pathways.

Collectively, these findings indicate that HPV-16 E6/E7 expression modulates hypoxia- and irradiation-driven transcriptional adaptations, leading to distinct stress-response programs in isogenic HNSCC cells.

### Tumor-level validation of transcriptional programs identified in HNSCC models

To confirm the effectiveness of hypoxic exposure in our experimental system, we first validated hypoxia pathway activation at the protein level. Western blot analysis of cell lysates from FaDu and 2A3 cells cultured under hypoxic and normoxic conditions demonstrated robust induction of the hypoxia marker CA IX under hypoxia (Supplementary file 2, Figure S2). In contrast, HIF-1α protein levels were not detectably increased under these conditions, which is consistent with previous reports showing transient stabilization of HIF-1α followed by its decline during prolonged hypoxic exposure^26,27^.

This observation was further supported by our transcriptomic data (Supplementary file 2, Table ST1), which confirmed robust activation of a canonical hypoxia-associated transcriptional program in both cell lines. Differential expression analysis revealed significant upregulation of established HIF-1 target genes involved in metabolic adaptation (*SLC2A1/3*,* HK2*,* PDK1*,* LDHA*), survival and autophagy (*BNIP3*,* BNIP3L*,* DDIT4*), extracellular matrix remodeling (*P4HA1*,* PLOD2*,* LOXL2*), and angiogenic signaling (*ADM*,* ANGPTL4*). Notably, strong induction of *CA9* and *NDRG1* further supports effective hypoxic exposure at the transcriptional level. While the core hypoxic response was conserved, 2A3 cells exhibited relatively stronger induction of extracellular matrix remodeling genes, whereas FaDu cells showed a more pronounced metabolic adaptation signature.

To determine whether the transcriptional programs identified under hypoxia and irradiation in our experimental models are reflected in patient tumors, we performed validation analyses using publicly available HNSCC datasets (*n* = 528; *n* = 279) accessed via cBioPortal and^28^. A targeted panel of 24 candidate genes (Supplementary file 3) was selected based on key biological processes identified in our transcriptomic analyses, including cell-cycle regulation and DNA damage response (e.g. *PRKDC*,* STAG1*,* MCM4*), apoptosis signaling (*APAF1*,* BIRC5*), cytokine and hypoxia-associated responses (*SERPINE1*,* IL24*,* CXCR6*,* CCL26*,* MIF*), and TGF-β/BMP pathway remodeling (*TGFBR2*,* SMAD3*,* BMP6*).

Comparison of HPV-positive and HPV-negative tumors revealed concordant differential expression patterns for several genes identified *in vitro*, including *SERPINE1*,* IL24*,* CCL26*,* CXCR6*,* TGFBR2*,* PRKDC*,* STAG1*, and *MCM4*, supporting the biological relevance of hypoxia-associated transcriptional responses observed in our experimental models (Supplementary file 3, HPVpos_vs_HPVneg).

To explore the association with metastatic behavior, we compared primary tumors without nodal involvement and tumors with pathological lymph node metastases (Supplementary file 3, Npos_vs_N0). This analysis identified a metastasis-associated transcriptional program characterized predominantly by suppression of epithelial differentiation-related genes in lymph node-positive tumors, consistent with increased tumor plasticity and invasive potential. Within the candidate panel, *GDF11* showed significant association with nodal metastatic status, while *SERPINE1* and *CCL26* exhibited consistent directional trends. Although effect sizes in this analysis were modest, this is expected given that both groups represent primary tumors differing in metastatic propensity rather than direct comparison of primary and metastatic lesions.

### Hypoxia increases doubling time and limits proliferation in HNSCC cells

**Fig. 2:**
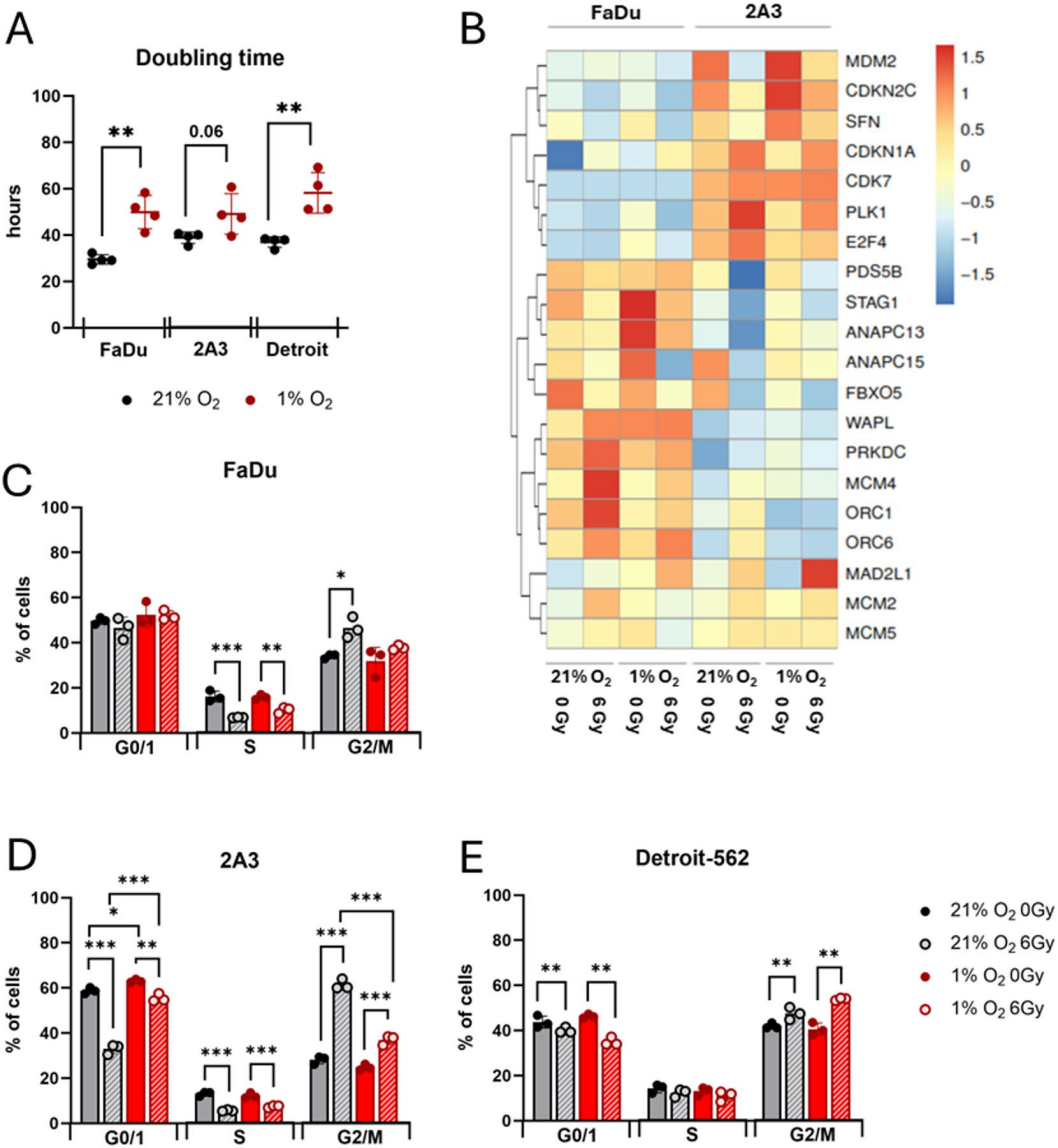
Proliferation and cell cycle regulation under different oxygen conditions and following gamma-irradiation. (**A**) FaDu, 2A3, and Detroit-562 cell lines were cultured under normoxic (21% O_2_) or hypoxic (1% O_2_) conditions. Cell numbers were determined 48 and 72 h after plating, and doubling times were calculated. Data represent the mean ± SD from four independent experiments (*n* = 4). Statistical analysis was performed using an unpaired t-test. (**B**) Heatmap of the top 20 DEGs involved in cell cycle regulation. Averaged data from three independent experiments are shown. The color bar is showing the values of z-score for each gene after library size normalization via DESeq2 software. Cell cycle distribution was analyzed 24 h after 6 Gy gamma-irradiation in (**C**) FaDu, (**D**) 2A3, and (**E**) Detroit-562 cells cultured under normoxic or hypoxic (1% O_2_) conditions. Results are presented as the percentage of cells in G0/1, S, or G2/M phases. Data represent the mean ± SD from three independent experiments (*n* = 3). Statistical analysis was performed using two-way ANOVA with Tukey’s multiple comparisons test. (*p < 0.05, **p < 0.01, ***p < 0.001).

Because hypoxia is known to suppress proliferation^29^ and thereby influence radiation sensitivity, we next examined how reduced oxygen tension affects growth kinetics and doubling time in tested HNSCC cell lines. Under normoxia, 2A3 cells (Fig. 2A) exhibited baseline doubling times (DT) comparable to Detroit-562, whereas the shorter DT of FaDu cells suggest higher proliferative activity. Next, we assessed the growth kinetics of HNSCC cell lines under hypoxia. As shown in Fig. 2A, all tested cell lines demonstrated increased DT under hypoxia, with FaDu and Detroit-562 showing significantly delayed proliferation, while 2A3 cells exhibited a near-significant change (*p* = 0.06). Notably, 2A3 cells displayed growth exhaustion, failing to proliferate beyond two weeks during prolonged hypoxia, whereas FaDu and Detroit-562 maintained stable growth (data not shown). Consequently, 2A3 cells were cultured under hypoxia for up to 14 days and re-established from ambient conditions before each new experiment. Collectively, these findings demonstrate that reduced oxygen availability limits HNSCC cell proliferation across different biological backgrounds, providing a functional context for subsequent radiation‑response analyses.

### Hypoxia limits radiation-induced cell-cycle arrest in a cell-dependent manner in HNSCC

Given that radiation efficacy is closely associated with cell‑cycle phase distribution and that hypoxia can alter cell‑cycle dynamics, we investigated how oxygen tension influences radiation‑induced cell‑cycle redistribution in HNSCC cells. We assessed transcriptomic alterations and cell‑cycle phase distribution 24 h after gamma‑irradiation in cells maintained under normoxic or hypoxic conditions (Fig. 2B–E).

Based on the transcriptomic analysis (Fig. 2B), 2A3 cells—unlike FaDu—showed, under hypoxia, increased expression of cell-cycle negative regulators (*MDM2*, *CDKN2C*). In contrast, FaDu showed increased expression of *STAG1*, *ANAPC13*/*15*, and *WAPL*, which are positive regulators of the cell cycle. Gamma-irradiation under ambient conditions differentially affected the DEGs in both cell lines: in FaDu, we observed increased expression of *WAPL*, *PRKDC*, *MCM4*, and *ORC1/6*, and the effects of irradiation on *PRKDC*, *MCM4*, and *ORC1* genes were mitigated under hypoxia; in 2A3 upregulation of *CDK7*, *PLK1*, and *E2F4* was detected. On the other hand, *PDS5B*, *STAG1*, and *ANAPC13/15* were downregulated in 2A3 under the same conditions. This effect was less evident in 2A3 irradiated under hypoxia.

All tested cell lines exhibited significant G2/M arrest after irradiation under normoxic conditions (Fig. 2C–E), with the most pronounced response observed in 2A3 cells. Under hypoxic conditions, this irradiation‑induced G2/M arrest was attenuated in FaDu cells but remained evident in 2A3 and Detroit‑562 cells, suggesting altered hypoxia-dependent modulation of radiation-induced cell-cycle arrest in FaDu cells. Notably, 2A3 cells displayed a significant hypoxia‑induced reduction of G2/M arrest after irradiation. In both FaDu and 2A3 cells, irradiation resulted in a significant reduction of the S‑phase fraction, independent of oxygen conditions (Fig. 2C, D). In contrast, 2A3 and Detroit‑562 cells showed a concomitant decrease in the G0/G1 fraction following irradiation. FaDu cells, however, exhibited no significant changes in the G0/G1 phase under either normoxic or hypoxic conditions (Fig. 2C–E).

Together, these data demonstrate that hypoxia differentially modulates radiation‑induced cell‑cycle arrest in HNSCC cells in a cell‑line‑dependent manner.

### Hypoxia modulates radiation-induced apoptotic signaling in isogenic HNSCC cells

To determine whether hypoxia modifies radiation‑induced apoptotic responses in HNSCC cells, we analyzed caspase‑3 activation and apoptosis‑related transcriptional changes following irradiation under normoxic and hypoxic conditions, with particular emphasis on the isogenic FaDu and 2A3 cell lines.

**Fig. 3:**
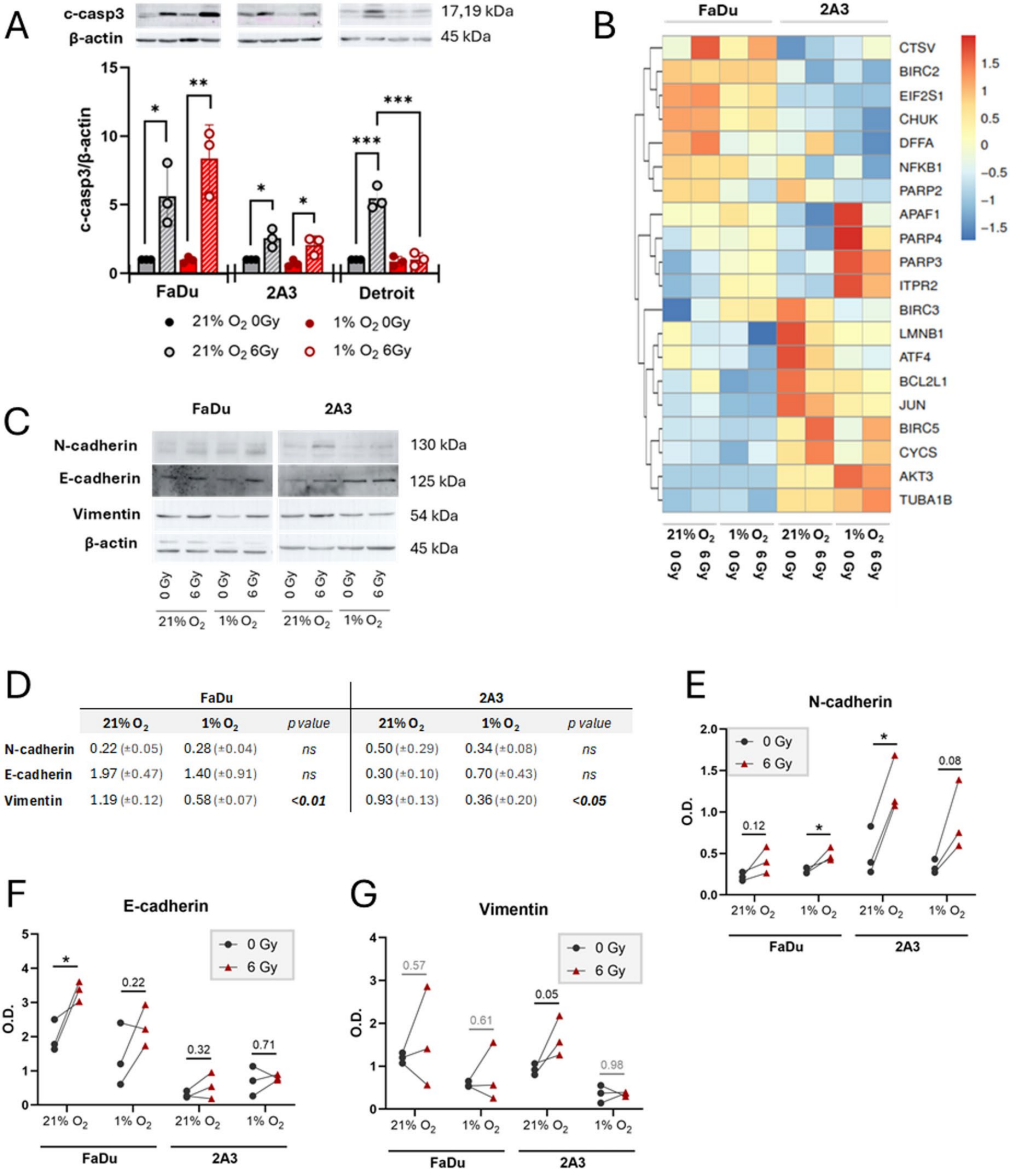
Effects of hypoxia and gamma-irradiation on apoptosis and EMT in HNSCC. (**A**) Cleaved caspase-3 levels in FaDu, 2A3, and Detroit-562 cells cultured under normoxic (21% O_2_) or hypoxic (1% O_2_) conditions, assessed 48 h after 6 Gy gamma-irradiation. Results are shown as fold change relative to non-irradiated normoxic controls. Data represent mean ± SD from three independent experiments (*n* = 3). Statistical analysis was performed using two-way ANOVA with Tukey’s multiple comparisons test. (**B**) Heatmap of the top 20 DEGs involved in apoptotic cell death. Averaged data from three independent experiments are shown. The color bar is showing the values of z-score for each gene after library size normalization via DESeq2 software. (**C**) Representative western blots of N-cadherin, E-cadherin, and vimentin in FaDu and 2A3 cells. (**D**) Quantification of protein levels expressed as mean optical density (O.D.) ± SD, normalized to β-actin, in FaDu and 2A3 cells under normoxic or hypoxic conditions. (**E**–**G**) Effects of hypoxia and irradiation on N-cadherin, E-cadherin, and vimentin levels in FaDu and 2A3 cells under normoxic and hypoxic conditions. Data represent mean ± SD from three independent experiments (*n* = 3). Statistical analysis was performed using unpaired t-test. (*p < 0.05, **p < 0.01, ***p < 0.001). Representative western blots (**A**,**C**) are shown. Images were cropped vertically to display only the relevant samples and horizontally to display the target protein band, with molecular weight indicated. Blots derived from different membranes are juxtaposed with clear spacing to indicate separate membranes. Original uncropped western blot membranes corresponding to these panels are provided in Supplementary file 4.

Cleaved caspase-3 levels (48 h after irradiation) increased under ambient conditions (Fig. 3A). In FaDu and 2A3 cells, this increase was significant under both normoxia and hypoxia, whereas in Detroit-562, cleaved caspase-3 was not elevated under hypoxia compared to normoxia (Fig. 3A).

Transcriptomic analysis (Fig. 3B; 24 h after irradiation) revealed that FaDu cells have lower baseline expression of anti-apoptotic genes (*BIRC3/5*, *BCL2L1* and *AKT3*) and higher expression of pro-apoptotic effectors (*EIF2S1*, *DFFA* and *APAF-1*) compared to 2A3. Under hypoxia (1% O_2_), FaDu exhibited downregulation of pro-apoptotic mediators (*DFFA*, *CYCS*,* PARP2*), while most anti-apoptotic genes remained unchanged (*BIRC2/5*, *AKT3*). On the other hand, 2A3 showed decreased expression of both anti/pro-apoptotic genes (*BIRC3*,* BCL2L1*, and *DFFA*, *CYCS*). Following irradiation under normoxia, FaDu showed mild upregulation of pro-apoptotic genes (*EIF2S1*, *DFFA*). In 2A3 cells, irradiation downregulated anti-apoptotic genes (*BIRC3*, *BCL2L1*). These effects were attenuated under hypoxia.

These results indicate that hypoxia modulates radiation‑induced apoptotic signaling in HNSCC cells in a cell‑line‑dependent manner.

### Hypoxia and irradiation differentially modulate EMT marker expression in HNSCC

Because hypoxia and irradiation have both been implicated in epithelial–mesenchymal plasticity, we next evaluated the effects of these stressors on the expression of canonical epithelial–mesenchymal transition (EMT) markers in HNSCC cell lines (Fig. 3C–G).

Hypoxia significantly reduced vimentin in FaDu and 2A3, while N-cadherin and E-cadherin remained unchanged (Fig. 3C, D). Irradiation further modulated EMT markers (Fig. 3E–G): N-cadherin increased in FaDu under hypoxia and in 2A3 under normoxia; E-cadherin was upregulated in FaDu under normoxia; and vimentin showed variable responses, with 2A3 trending toward upregulation. Overall, hypoxia primarily downregulated vimentin level, while irradiation induced N-cadherin and E-cadherin expression in FaDu, indicating cell line–specific EMT modulation with potential implications for invasiveness. These findings indicate that HPV-16 E6/E7 expression contributes to differential modulation of EMT marker expression in response to hypoxia and irradiation in HNSCC cells.

In both FaDu and 2A3 cells, irradiation under ambient air downregulated canonical (transforming growth factor β) TGF-β components such as *TGFB2*, *TGFBR2*, *SP1*, and *SMAD3*, while simultaneously upregulating bone morphogenic protein (BMP) and its ligands e.g. *BMP6* in FaDu, *GDF11* in 2A3. This pattern suggests a shift away from canonical TGF-β/SMAD signaling, which is often associated with EMT and tumor progression, toward BMP-driven or inhibitory networks potentially restricting EMT and favoring differentiation. Hypoxia alone further reinforced this trend, suppressing *TGFB1/2*, *SMAD5/6*, and *PITX2* while increasing *SMAD3*, *INHBA*, *FST*, and *SMURF2*, consistent with remodeling of TGF-β signaling and induction of antagonistic feedback loops. However, these effects were cell-type specific (Supplementary file 2, Figure S3).

### Hypoxia- and irradiation-induced changes in cytokine expression and release

**Fig. 4:**
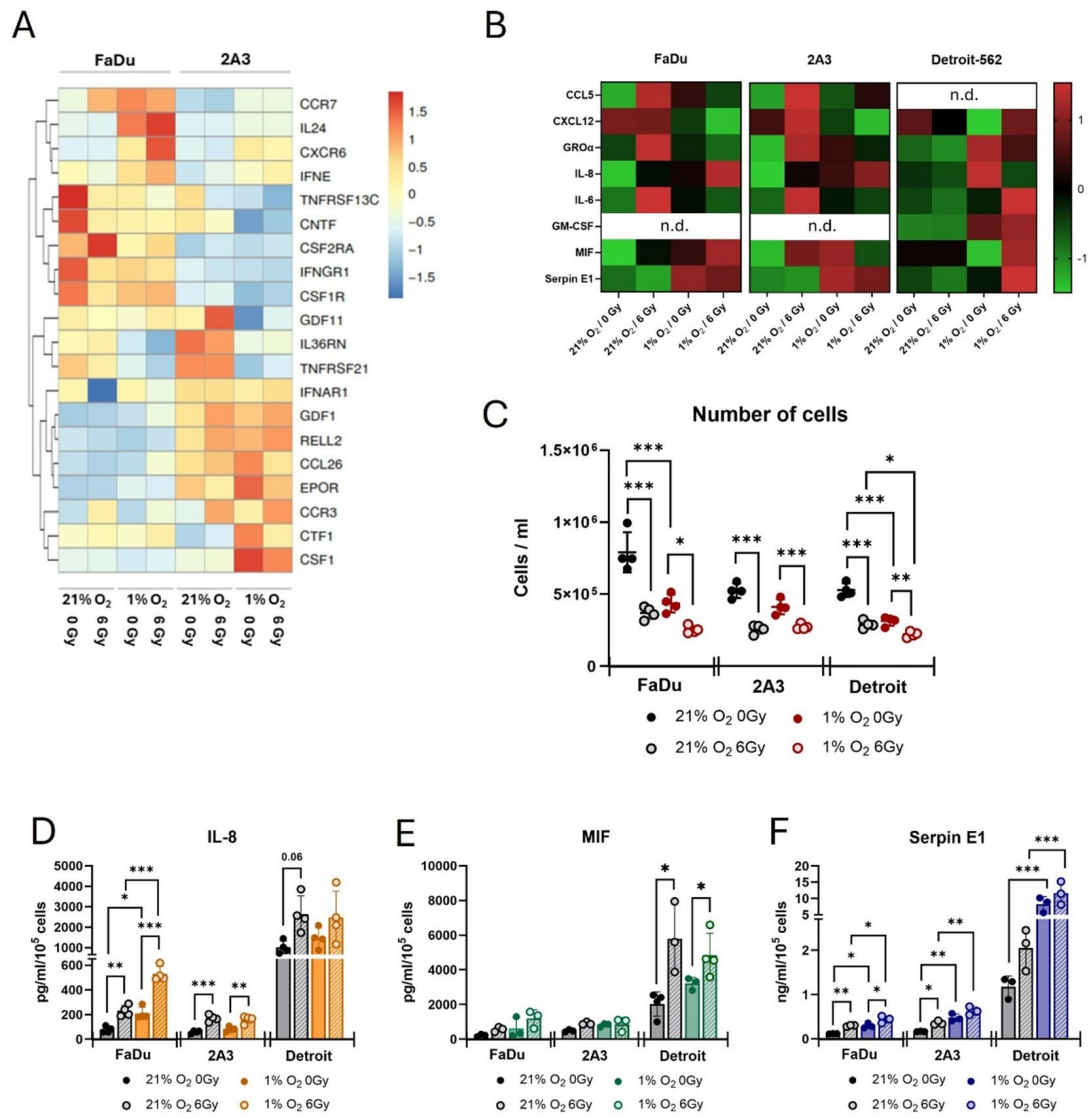
Effects of hypoxia and gamma-irradiation on cytokine and chemokine release in HNSCC. (**A**) Heatmap of the top 20 DEGs. Averaged data from three independent experiments are shown. The color bar is showing the values of z-score for each gene after library size normalization via DESeq2 software. (**B**) Cytokine and chemokine production in response to hypoxia, gamma-irradiation, and their combination. Conditioned media from FaDu, 2A3, and Detroit-562 cells were collected 48 h after gamma-irradiation under normoxic or hypoxic conditions. Media from three independent experiments per cell line were pooled for analysis. Results are presented as z-scores of log₁₀-transformed normalized data. n.d. = not detected. (**C**) Cell numbers of FaDu, 2A3, and Detroit-562 cultured under normoxic (21% O_2_) or hypoxic (1% O_2_) conditions, assessed 48 h after 6 Gy gamma-irradiation. Data represent mean ± SD from four independent experiments (*n* = 4). Statistical analysis was performed using two-way ANOVA with Tukey’s multiple comparisons test. Levels of IL-8 (**D**), MIF (**E**), and Serpin E1 (**F**) in supernatants from FaDu, 2A3, and Detroit-562 cells cultured under normoxic or hypoxic conditions, assessed 48 h after gamma-irradiation. Concentrations were normalized to cell numbers per condition. Data represent mean ± SD from three to four independent experiments (*n* = 3–4). Statistical analysis was performed using two-way ANOVA with Tukey’s multiple comparisons test. (*p < 0.05, **p < 0.01, ***p < 0.001).

As hypoxia and irradiation can remodel the tumor microenvironment through altered cytokine and chemokine signaling, we investigated how these conditions affect inflammatory mediator expression at both the transcriptomic and protein levels in HNSCC cells. Transcriptomic changes were first assessed to identify hypoxia‑ and irradiation‑responsive cytokine and chemokine genes (Fig. 4A), followed by protein‑level validation using cytokine arrays and ELISA (Fig. 4B–F).

In FaDu cells, hypoxia slightly downregulated *CSF1*, *CSF1R*, and *CSF2RA*, indicating reduced GM-CSF signaling, and upregulated *IL24* and *CXCR6*, favoring immune activation and potential tumor suppression. Upon irradiation under hypoxia, *IL24* and *CXCR6* further increased in FaDu, accompanied by only modest *CSF2RA* induction and a small rise in invasion-associated markers such as *CCL26* and *GDF11*, suggesting a shift toward a more immune-permissive yet more aggressive phenotype. In 2A3 cells, hypoxia alone induced *CXCR6* and also upregulated *CSF1*, *EPOR*, and *CCL26*, supporting a more pro-tumorigenic, invasive profile. Under hypoxic irradiation, 2A3 partially reversed this progression-associated profile, with reductions in *CSF1*, *EPOR*, and *CCL26*, indicating partial normalization of the hypoxia-driven invasive signature. Strikingly, the combination of hypoxia and irradiation produced the most pronounced effects, upregulating *IL24*,* IFNE* in FaDu, and chemokine receptors (*CXCR6* in FaDu, and *CCR3* in 2A3), while strongly repressing *SP1*,* TGFB2*,* SMAD1/6*, and *PITX2* (Figs. 4A, Supplementary file 2, Figure S3).

At the protein level, as assessed by cytokine array analysis (Fig. 4B), the cell lines differed in both detectable cytokine and chemokine profiles and responsiveness to hypoxia, irradiation, or their combination. FaDu and 2A3 produced CCL5, which was absent in Detroit-562. In contrast, Detroit-562 secreted high levels of GM-CSF, not detected in the other two lines. Under normoxia, FaDu and 2A3 exhibited similar cytokine and chemokine induction after irradiation (Fig. 4B), including increased CCL5, CXCL12, GROα, IL-8, and IL-6. Under hypoxia, MIF and Serpin E1 were elevated, while Detroit-562 showed broader upregulation across nearly all detectable analytes. Based on these findings, signal‑to‑background ratios, and their well‑established involvement in hypoxia‑ and irradiation‑responsive tumor biology, IL‑8, MIF, and Serpin E1 were selected for subsequent ELISA quantification.

To quantitatively validate array‑based findings and assess condition‑specific cytokine release with higher sensitivity, we performed targeted ELISA analyses of IL‑8, MIF, and Serpin E1 in conditioned media normalized to cell number. Cell numbers were therefore determined prior to cytokine quantification. A marked reduction was observed in non-irradiated hypoxic controls at 48 h post-sham irradiation, with significant decreases in FaDu and Detroit-562 cells (Fig. 4C). Similarly, 6 Gy irradiation significantly reduced cell counts in all cell lines, more strongly under normoxia, while irradiation under hypoxia caused a smaller yet significant decline. Among the three cell lines, only Detroit-562 showed a statistically significant difference between normoxic and hypoxic irradiation (Fig. 4C).

IL-8 production in FaDu cells was dependent on both oxygen and radiation, with a significant interaction effect (Fig. 4D). In contrast, 2A3 cell line exhibited a radiation-dependent increase in IL-8, with no significant effect of oxygen, a trend that was also observed in Detroit-562 cells (Fig. 4D). Notably, IL-8 production in 2A3 was 2–3 times lower than in FaDu, whereas Detroit-562 cells produced IL-8 at levels approximately an order of magnitude higher than FaDu under comparable conditions (Fig. 4D).

MIF levels were modestly elevated in FaDu and 2A3 cells in response to hypoxia or irradiation, although these changes did not reach statistical significance (Fig. 4E). In contrast, gamma-irradiation significantly increased MIF production in Detroit-562 cells (Fig. 4E). While FaDu and 2A3 cells exhibited comparable maximum MIF levels, Detroit-562 cells secreted 4- to 7-fold higher amounts under the same conditions (Fig. 4E).

Serpin E1 levels increased significantly in response to hypoxia across all three HNSCC cell lines (Fig. 4F). In FaDu and 2A3 cells, irradiation under ambient air conditions also led to a significant increase in Serpin E1 levels, an effect that was not observed in Detroit-562 cells. Notably, in both FaDu and 2A3, irradiation under hypoxic conditions resulted in a significantly greater additive effect compared to irradiation under normoxia.

IL-8, MIF, and Serpin E1 levels 24 h after gamma-irradiation, illustrating time-dependent release patterns, are shown in Supplementary file 2, Figure S4.

These results demonstrate that hypoxia and irradiation differentially regulate cytokine and chemokine release in HNSCC in a cell‑line‑dependent manner, with metastatic Detroit‑562 cells exhibiting markedly higher secretion levels than the isogenic FaDu and 2A3 models.

## Discussion

HPV-associated HNSCC constitute a biologically distinct subgroup with a generally favorable prognosis, yet the determinants of their differential treatment response—particularly under hypoxia—remain incompletely defined. Because hypoxia is prevalent in HNSCC and modulates radiobiology and inflammatory signaling, we examined how oxygen tension (ambient vs. 1% O_2_) shaped radiation responses in HNSCC models differing in HPV oncogene status. To this end, we integrated analyses of proliferation, cell-cycle distribution, and apoptosis with transcriptomic profiling and cytokine and chemokine secretion, using isogenic FaDu‑derived cells with or without HPV‑16 E6/E7 expression and the metastatic Detroit‑562 cell line to capture effects related to tumor origin.

Increased doubling time under hypoxia aligned with reports that hypoxia prolongs the lag phase of HNSCC proliferation, even if subsequent growth rates remain similar^30^. Hypoxia slowed proliferation in all cell lines, but only 2A3 showed significant G0/G1 accumulation, indicating a distinct adaptation. In KB-3-1 cells (a subclone of HeLa cells infected with HPV-18), chronic hypoxia reduced proliferation with G-phase arrest, suggesting that hypoxia blocks G–S transition^31^, whereas in other HNSCC models, hypoxia induced cyclin D1 expression to promote G1–S transition^32^. These alterations in cell-cycle progression are consistent with the transcriptional changes observed in our analyses, reflecting a broader metabolic and regulatory reprogramming aimed at conserving energy and supporting cell survival under oxygen limitation. In FaDu cells, suppression of energy-demanding pathways including glucuronidation, xenobiotic metabolism, and retinoid processing suggests a metabolic reprogramming in response to oxygen deprivation, in line with hypoxia-driven shifts mediated by HIF-1α^33^. In 2A3 cells, downregulation of genes involved in epithelial dynamics and tissue development may reflect broader inhibition of morphogenetic programs under hypoxic stress^34^. Overall, these findings illustrate how hypoxia drives the repression of energetically costly and differentiation-related pathways in a cell line-specific manner.

At baseline, HPV-negative and HPV-positive HNSCC cell lines, including 2A3, showed no differences in cell-cycle distribution^35,36^. Consistent with our results, gamma-irradiation induced G2/M arrest in both, with stronger and longer arrest in HPV-positive cells^35–38^, though some reported this only in HPV-positive HNSCC^8^. We also observed S-phase reduction in FaDu and 2A3 cells post-irradiation, irrespective of E6/E7 expression, contrasting Arenz et al., who reported it only in HPV-negative cells^37^.

Few studies have examined radiation–cell cycle interactions under hypoxia in HNSCC. In other cancers, hypoxia can impair radiation-induced G2/M arrest^39^. Ha et al. showed that gamma-irradiation failed to induce G2/M arrest under chemical hypoxia^40^. Similarly, U87MG-E7 glioma cells lost G2/M arrest under hypoxia due to HPV-16 E7^41^. Consistent with this, E6/E7 expressing 2A3 cells showed reduced radiation-induced G2/M arrest under hypoxia with persistent G0/G1 accumulation. Detroit-562 cells, however, maintained G2/M arrest regardless of oxygen, suggesting resistance to hypoxia-induced modulation.

These findings underscore cancer cell line–specific interactions between hypoxia, irradiation, and expression of HPV-16 E6/E7 oncogenes in shaping cell cycle responses. Such functional differences likely originate from underlying transcriptional adaptations, as our data reveal distinct molecular programs activated under combined hypoxia and irradiation. Specifically, gamma-irradiation under hypoxic conditions not only enhances the repression of energy-demanding pathways but also drives divergent transcriptional responses such as stress-induced plasticity in FaDu cells and morphogenetic activation in 2A3 cells, both of which may influence tumor progression and radiotherapy outcomes. Similar processes involving metabolic reprogramming and morphogenetic activation have also been reported in other epithelial malignancies e.g. pancreatic cancer, where they contribute to enhanced adaptability and invasive behavior^42^.

In addition to cell cycle regulation, gamma-irradiation induced cleavage of caspase-3—a key effector caspase in both intrinsic and extrinsic apoptotic pathways—in all three HNSCC cell lines under ambient conditions. Notably, cleaved caspase-3 did not increase following irradiation under hypoxia in Detroit-562 cells, in contrast to FaDu and 2A3, which may reflect a shift toward checkpoint arrest rather than apoptosis (G2/M arrest preserved in hypoxia). Compared with HPV-negative cells, HPV-positive cells at ambient conditions showed increased caspase-3/7 activity after irradiation^35,43^. However, irradiation induced comparable apoptosis in FaDu and 2A3^36^. Long-term hypoxia was not associated with increased apoptosis in HPV-18–positive cancer cells after irradiation compared to normoxia^31^. Interestingly, in vivo, the hypoxic fraction of HPV-positive tumors decreased after irradiation, an effect not observed in HPV-negative tumors^44^. Therefore, the differential response of HPV-positive and HPV-negative HNSCC to irradiation under hypoxia highlights the potential of pharmacological interventions, such as AKT inhibition, to facilitate stratified treatment strategies^45^. Together, these findings demonstrate that hypoxia modulates radiation response through distinct, cell‑line‑dependent mechanisms, with apoptosis‑mediated radioresistance associated with suppression of caspase‑3 activation evident in Detroit‑562 cells, while alternative non‑apoptotic outcomes (e.g., senescence, lysosome‑mediated cell death, autophagy, or mitotic catastrophe) are likely to contribute in FaDu‑derived models.

Because hypoxia and ionizing radiation profoundly remodel the tumor microenvironment by altering cytokine and chemokine signaling, we next examined how these stressors shape inflammatory mediator release in HNSCC. Although high pretreatment IL-8 has been associated with worse overall survival, it did not correlate with hypoxia staining^46^. In our work, IL-8 increased under hypoxia only in FaDu cells, whereas irradiation upregulated IL-8 across all lines; a significant hypoxia–irradiation interaction was observed only in FaDu. Detroit-562 cells secreted markedly higher IL-8 than FaDu and 2A3. Prior studies reported both increases and decreases in IL-8 after irradiation, regardless of HPV status^47,48^. Clinically, a subgroup of HPV-positive patients with elevated pretreatment IL-8 showed improved outcomes compared with HPV-negative patients^10^. However, baseline IL-8 was higher in patients who later progressed, irrespective of HPV status, and all progressing HPV-negative patients displayed elevations in this high-risk factor^49^. These findings emphasize the need for combined hypoxia- and HPV-based stratification and suggest that integrating cytokine and chemokine risk profiles with HPV status may improve identification of patients at elevated risk of progression. Moreover, it appears that cytokine regulation under combined hypoxic and irradiative stress is not limited to IL-8, but reflects a wider remodeling of TGF-β/BMP signaling, shaping both inflammatory and differentiation-related responses. These findings point toward an integrated stress response, in which cytokine induction is coupled to suppression of canonical TGF-β signaling and activation of interferon-driven and pro-inflammatory programs, consistent with features of immunogenic cell death^50^.

MIF expression increases during HNSCC progression, and serum levels are elevated in patients compared with healthy controls^51^. Hypoxia induces cellular MIF expression and HIF-1α–dependent secretion^52^, while ionizing radiation elevates MIF in cancer models^53^. Consistently, we observed radiation-induced MIF secretion in Detroit-562 cells but not in FaDu or 2A3. Intracellular MIF was higher in HPV-negative tumors than in HPV-positive ones, yet in vitro conditioned medium from HPV-positive lines contained more MIF than from HPV-negative lines^7^. Under hypoxia, HPV-negative lines released greater MIF than HPV-positive ones, likely reflecting higher baseline HIF-1α and constitutive secretion^7^. In our study, Detroit-562 (HPV-negative) cells secreted substantially more MIF than 2A3, with hypoxia producing only modest increases. Differences with prior work may reflect hypoxia severity and duration (0.1% O_2_ for 48 h vs. chronic 1% O_2_). Importantly, MIF also promotes neutrophil chemotaxis, underscoring its role in shaping the tumor microenvironment^54^.

Elevated Serpin E1 levels correlate with poor prognosis in HNSCC, likely due to its role in promoting migration and inhibiting apoptosis^55^. Both hypoxia and irradiation induce Serpin E1 expression in HNSCC cell lines, with hypoxia showing a stronger effect in FaDu cells^56^. We similarly observed hypoxia as the dominant driver but also detected irradiation-induced Serpin E1 release in FaDu and 2A3, consistent with reports at higher radiation doses^57^. In vivo, Serpin E1 correlated with local tumor control and was elevated in hypoxic xenografts^58^. Clinically, baseline plasma Serpin E1 levels and tumor Serpin E1 expression were significantly higher in HPV/p16-negative patients^10,59^. Previous analyses have shown that FaDu cells exhibit relatively low Serpin E1 mRNA expression compared with other HNSCC cell lines, underscoring marked inter‑cell‑line heterogeneity in basal Serpin E1 levels^55^. Notably, enforced expression of HPV‑16 E6/E7 did not alter baseline or condition‑dependent changes in Serpin E1 secretion in the 2A3 model. In contrast, Detroit‑562 cells (HPV‑negative, metastatic origin) displayed higher basal Serpin E1 production as well as a more pronounced induction following hypoxia and irradiation. Transcriptional levels of Serpin E1, together with MIF and VEGF, were increased in HNSCC lines under hypoxia. Interestingly, Serpin E1 expression returned to near baseline after reoxygenation, suggesting it as a candidate gene for the hypoxic tumor phenotype^60^. Together, these findings indicate that Serpin E1 is modulated by both hypoxia and irradiation, and its elevated expression—particularly under hypoxic and HPV-negative conditions—may drive treatment resistance, supporting its potential relevance as a candidate biomarker and therapeutic target in the context of stratified radiotherapy.

HPV‑positive HNSCC are associated with significantly improved survival outcomes compared with HPV‑negative disease, including better overall, disease‑specific, and progression‑free survival^61^. HPV‑positive HNSCC cells are generally more radiosensitive than HPV‑negative cells, a phenotype largely attributed to impaired DNA double‑strand break repair, persistent G2/M arrest and reduced clonogenic survival following irradiation^62^. For example, in HPV‑negative HNSCC, intact TGF‑β signaling promotes DNA damage repair proficiency and relative radioresistance, whereas HPV-associated suppression of TGF‑β signaling–attributed in part to viral oncogene activity (e.g. E6/E7)–impairs homologous recombination (e.g. ATM, FOXO3, BRCA1) and enhances sensitivity to radiotherapy^63^. While HPV E6/E7 primarily affect DNA damage response pathways, HPV E5 has been shown to enhance radiosensitivity through upregulation of CENPM and an increased proportion of cells in G2/M-phase^64^. Collectively, these findings highlight that HPV‑associated radiosensitivity reflects the combined effects of individual viral oncogenes versus the full HPV genomic context, underscoring that radiation responses observed in simplified in vitro models—such as E6/E7‑expressing isogenic cell lines—may not fully recapitulate the DNA damage/repair, cytokine, and TGF‑β–dependent phenotypes of bona fide HPV‑positive tumors, thereby representing a limitation for translational interpretation (below).

This study has several limitations that should be considered when interpreting the findings. First, although we employed an isogenic FaDu-derived model with enforced HPV-16 E6/E7 expression to interrogate HPV-associated mechanisms, this approach does not fully recapitulate the complexity of naturally occurring HPV-positive HNSCC, which are shaped by additional viral (e.g. episomal vs. integrated status^65^, genetic (e.g. mutation burden^66^, intrinsic DNA damage/repair capacity^62^, expression of distinct or combined viral oncogenes^63,64^, and microenvironmental factors (e.g. stromal components of the tumor and infiltration of innate and/or adaptive immune cells^67^. Moreover, the number of cell lines used per category was limited, precluding generalization of our observations to all HPV-positive or HPV-negative HNSCC. Accordingly, our conclusions should be interpreted as model- and cell-line-specific, and broader validation using larger panels of bona fide HPV-positive, HPV-negative, and metastatic HNSCC models, as well as in vivo systems, will be required. In addition, baseline DNA damage/replication stress levels and TGF‑β pathway activity were not systematically profiled across all models, which may further contribute to the observed heterogeneity in radiation responses.

To partially address the limitations inherent to in vitro systems, we complemented our experimental findings with tumor-level validation using TCGA HNSCC datasets. These analyses confirmed that several key genes and pathways identified under hypoxia and irradiation in our models are also differentially regulated in patient tumors, particularly in relation to HPV status and nodal metastatic dissemination. While such analyses cannot fully capture the complexity of the tumor microenvironment, they provide important orthogonal support for the biological relevance of our findings.

In addition, the use of a single hypoxic condition (1% O_2_) represents a defined experimental model and does not reflect the dynamic spatial and temporal oxygen gradients characteristic of physiological tumor hypoxia. As a result, the observed radiation responses under hypoxic conditions should be interpreted as reflecting this specific level of hypoxic stress rather than the full spectrum of hypoxia present within tumors. Finally, while we focused on a selected set of cytokines and apoptotic markers, broader cytokine networks and additional radiation‑induced cell fates, such as senescence or mitotic catastrophe, were not evaluated and warrant future investigation.

**Fig. 5:**
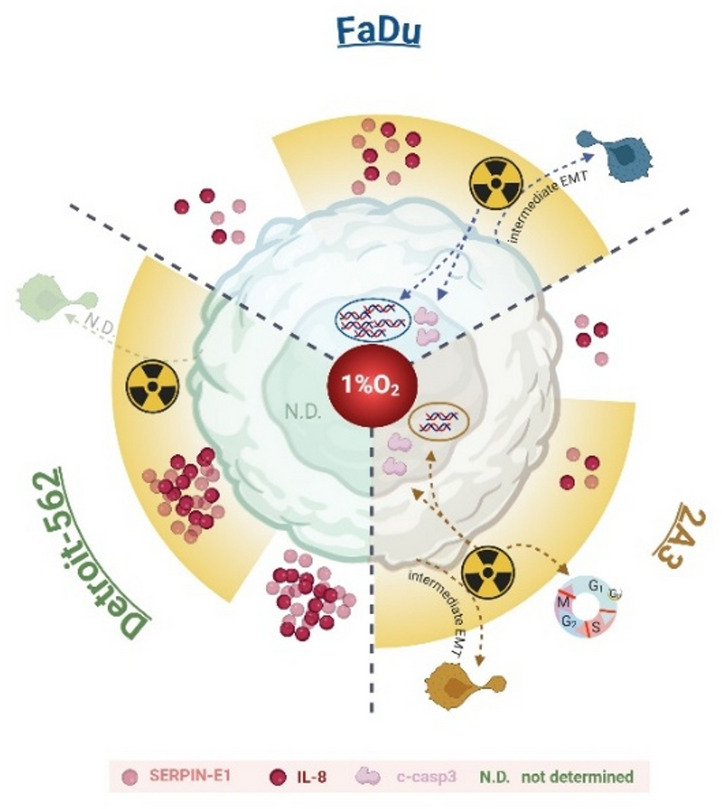
Schematic representation of the proposed interactions between hypoxia and gamma irradiation in the HNSCC cell lines studied. IL‑8 – interleukin 8; c‑casp3 – cleaved caspase‑3. Created in BioRender. Pereckova, J. (2026) https://BioRender.com/zavemf9.

## Conclusions

This study provides new mechanistic insights into how hypoxia modulates radiation responses in HNSCC cell lines. Using a multi-layered approach—including proliferation, cell-cycle profiling, apoptosis markers, transcriptomics, and cytokine and chemokine secretion—we showed that hypoxia attenuated radiation-induced G2/M arrest and caspase-3 activation in a cell line–dependent manner and altered secretion of IL-8, MIF, and Serpin E1 (Fig. 5). These findings highlight reduced therapeutic efficacy under low-oxygen conditions and underscore the importance of integrating tumor hypoxia with biological context, including HPV oncogene status and tumor origin, when considering future biomarker-guided and stratified radiotherapy strategies for HNSCC. Together, these results provide a foundation for developing targeted interventions to overcome hypoxia-driven treatment resistance.

## Supplementary Information

Below is the link to the electronic supplementary material.

Supplementary Material 1

Supplementary Material 2

Supplementary Material 3

Supplementary Material 4

Supplementary Material 5

## Data Availability

The NGS datasets generated and analyzed in this study will be made publicly available upon acceptance in the NCBI public database, http://www.ncbi.nlm.nih.gov/bioproject/1348988.

## References

[1] Hammond, E. M. et al. The meaning, measurement and modification of hypoxia in the laboratory and the clinic. *Clin. Oncol. (R. Coll. Radiol.)* **26**(5), 277–88. 10.1016/j.clon.2014.02.002 (2014). 24602562 10.1016/j.clon.2014.02.002

[2] Brown, J. M. Tumor hypoxia in cancer therapy. *Methods Enzymol.* **435**, 297–321. 10.1016/S0076-6879(07)35015-5 (2007). 17998060 10.1016/S0076-6879(07)35015-5

[3] Hauth, F., Toulany, M., Zips, D. & Menegakis, A. Cell-line dependent effects of hypoxia prior to irradiation in squamous cell carcinoma lines. *Clin. Transl. Radiat. Oncol.* **5**, 12–9. 10.1016/j.ctro.2017.06.001 (2017). 29594212 10.1016/j.ctro.2017.06.001 PMC5833923

[4] Machiels, J. P. et al. Squamous cell carcinoma of the oral cavity, larynx, oropharynx and hypopharynx: EHNS-ESMO-ESTRO Clinical practice guidelines for diagnosis, treatment and follow-up. *Ann. Oncol.* **31**(11), 1462–75. 10.1016/j.annonc.2020.07.011 (2020). 10.1016/j.annonc.2020.07.011 33239190

[5] Borras, J. M. et al. The impact of cancer incidence and stage on optimal utilization of radiotherapy: Methodology of a population based analysis by the ESTRO-HERO project. *Radiother. Oncol.* **116**(1), 45–50. 10.1016/j.radonc.2015.04.021 (2015). 26002304 10.1016/j.radonc.2015.04.021

[6] Johnson, D. E. et al. Head and neck squamous cell carcinoma. *Nat. Rev. Dis. Primers.* **6**(1), 92. 10.1038/s41572-020-00224-3 (2020). 33243986 10.1038/s41572-020-00224-3 PMC7944998

[7] Kindt, N. et al. Involvement of HPV infection in the release of macrophage migration inhibitory factor in head and neck squamous cell carcinoma. *J. Clin. Med.*10.3390/jcm8010075 (2019). 30634708 10.3390/jcm8010075 PMC6352225

[8] Rieckmann, T. et al. HNSCC cell lines positive for HPV and p16 possess higher cellular radiosensitivity due to an impaired DSB repair capacity. *Radiother. Oncol.* **107**(2), 242–6. 10.1016/j.radonc.2013.03.013 (2013). 23602369 10.1016/j.radonc.2013.03.013

[9] Sorensen, B. S. et al. Radiosensitivity and effect of hypoxia in HPV positive head and neck cancer cells. *Radiother. Oncol.* **108**(3), 500–5. 10.1016/j.radonc.2013.06.011 (2013). 23953409 10.1016/j.radonc.2013.06.011

[10] Brondum, L. et al. Plasma proteins as prognostic biomarkers in radiotherapy treated head and neck cancer patients. *Clin. Transl. Radiat. Oncol.* **2**, 46–52. 10.1016/j.ctro.2017.01.001 (2017). 29658000 10.1016/j.ctro.2017.01.001 PMC5893530

[11] Trellakis, S. et al. Polymorphonuclear granulocytes in human head and neck cancer: Enhanced inflammatory activity, modulation by cancer cells and expansion in advanced disease. *Int. J. Cancer* **129**(9), 2183–93. 10.1002/ijc.25892 (2011). 21190185 10.1002/ijc.25892

[12] Kalayci Yigin, A., Azzawri, A., Ozturk, K., Cora, T. & Seven, M. Determination of cytokine profile and associated genes of the signaling pathway in HNSCC. *J. Recept. Signal Transduct. Res.* **42**(5), 462–468. 10.1080/10799893.2021.2013888 (2022). 34886759 10.1080/10799893.2021.2013888

[13] Linkov, F. et al. Early detection of head and neck cancer: Development of a novel screening tool using multiplexed immunobead-based biomarker profiling. *Cancer Epidemiol. Biomarkers Prev.* **16**(1), 102–7. 10.1158/1055-9965.EPI-06-0602 (2007). 17220337 10.1158/1055-9965.EPI-06-0602

[14] Harris, M. et al. Radioimmunotherapy of experimental head and neck squamous cell carcinoma (HNSCC) with E6-specific antibody using a novel HPV-16 positive HNSCC cell line. *Head Neck Oncol.* **3**(1), 9. 10.1186/1758-3284-3-9 (2011). 21314983 10.1186/1758-3284-3-9 PMC3046925

[15] Perecko, T., Pereckova, J., Hoferova, Z. & Falk, M. Cell-type specific anti-cancerous effects of nitro-oleic acid and its combination with gamma irradiation. *Biol. Chem.* **405**(3), 177–87. 10.1515/hsz-2023-0150 (2024). 37712609 10.1515/hsz-2023-0150

[16] Pastorekova, S., Zavadova, Z., Kostal, M., Babusikova, O. & Zavada, J. A novel quasi-viral agent, MaTu, is a two-component system. *Virology* **187** (2), 620–626. 10.1016/0042-6822(92)90464-z (1992). 1312272 10.1016/0042-6822(92)90464-z

[17] Svastova, E. et al. Carbonic anhydrase IX interacts with bicarbonate transporters in lamellipodia and increases cell migration via its catalytic domain. *J. Biol. Chem.* **287**(5), 3392–402. 10.1074/jbc.M111.286062 (2012). 22170054 10.1074/jbc.M111.286062 PMC3270993

[18] Yates, A. D. et al. Ensembl 2020. *Nucleic Acids Res.* **48** (D1), D682. 10.1093/nar/gkz966 (2020). 31691826 10.1093/nar/gkz966 PMC7145704

[19] Kim, D., Paggi, J. M., Park, C., Bennett, C. & Salzberg, S. L. Graph-based genome alignment and genotyping with HISAT2 and HISAT-genotype. *Nat. Biotechnol.* **37**(8), 907–15. 10.1038/s41587-019-0201-4 (2019). 31375807 10.1038/s41587-019-0201-4 PMC7605509

[20] Liao, Y., Smyth, G. K. & Shi, W. featureCounts: An efficient general purpose program for assigning sequence reads to genomic features. *Bioinformatics* **30**(7), 923–30. 10.1093/bioinformatics/btt656 (2014). 24227677 10.1093/bioinformatics/btt656

[21] Love, M. I., Huber, W. & Anders, S. Moderated estimation of fold change and dispersion for RNA-seq data with DESeq2. *Genome Biol.* **15**(12), 550. 10.1186/s13059-014-0550-8 (2014). 25516281 10.1186/s13059-014-0550-8 PMC4302049

[22] Heberle, H., Meirelles, G. V., da Silva, F. R., Telles, G. P. & Minghim, R. InteractiVenn: A web-based tool for the analysis of sets through Venn diagrams. *BMC. Bioinformatics* **16**(1), 169. 10.1186/s12859-015-0611-3 (2015). 25994840 10.1186/s12859-015-0611-3 PMC4455604

[23] Ge, S. X., Son, E. W. & Yao, R. iDEP: An integrated web application for differential expression and pathway analysis of RNA-Seq data. *BMC Bioinformatics* **19**(1), 534. 10.1186/s12859-018-2486-6 (2018). 10.1186/s12859-018-2486-6 PMC6299935 30567491

[24] Elizarraras, J. M. et al. WebGestalt 2024: Faster gene set analysis and new support for metabolomics and multi-omics. *Nucleic Acids Res.* **52**(W1), W415–W21. 10.1093/nar/gkae456 (2024). 38808672 10.1093/nar/gkae456 PMC11223849

[25] Kanehisa, M. & Goto, S. KEGG: Kyoto encyclopedia of genes and genomes. *Nucleic Acids Res.* **28**(1), 27–30. 10.1093/nar/28.1.27 (2000). 10592173 10.1093/nar/28.1.27 PMC102409

[26] Obacz, J., Pastorekova, S., Vojtesek, B. & Hrstka, R. Cross-talk between HIF and p53 as mediators of molecular responses to physiological and genotoxic stresses. *Mol. Cancer* **12**(1), 93. 10.1186/1476-4598-12-93 (2013). 23945296 10.1186/1476-4598-12-93 PMC3844392

[27] Uchida, T. et al. Prolonged hypoxia differentially regulates hypoxia-inducible factor (HIF)-1alpha and HIF-2alpha expression in lung epithelial cells: implication of natural antisense HIF-1alpha. *J. Biol. Chem.* **279** (15), 14871–14878. 10.1074/jbc.M400461200 (2004). 14744852 10.1074/jbc.M400461200

[28] Cancer Genome Atlas N. Comprehensive genomic characterization of head and neck squamous cell carcinomas. *Nature* **517** (7536), 576–582. 10.1038/nature14129 (2015). 25631445 10.1038/nature14129 PMC4311405

[29] Hubbi, M. E. & Semenza, G. L. Regulation of cell proliferation by hypoxia-inducible factors. *Am. J. Physiol. Cell Physiol.* **309**(12), C775–C782. 10.1152/ajpcell.00279.2015 (2015). 26491052 10.1152/ajpcell.00279.2015 PMC4683214

[30] Shinohara, Y. et al. Hypoxically cultured cells of oral squamous cell carcinoma increased their glucose metabolic activity under normoxic conditions. *PLoS One*. **16** (10), e0254966. 10.1371/journal.pone.0254966 (2021). 34679081 10.1371/journal.pone.0254966 PMC8535375

[31] Cuisnier, O. et al. Chronic hypoxia protects against gamma-irradiation-induced apoptosis by inducing bcl-2 up-regulation and inhibiting mitochondrial translocation and conformational change of bax protein. *Int. J. Oncol.* **23** (4), 1033–1041 (2003). 12963983

[32] Yin, X. et al. Metformin sensitizes hypoxia-induced gefitinib treatment resistance of HNSCC via cell cycle regulation and EMT reversal. *Cancer Manag. Res.* **10**, 5785–5798. 10.2147/CMAR.S177473 (2018). 30510448 10.2147/CMAR.S177473 PMC6250113

[33] Besso, M. J. et al. Transcriptomic and epigenetic landscape of nimorazole-enhanced radiochemotherapy in head and neck cancer. *Radiother. Oncol.* **199**, 110348. 10.1016/j.radonc.2024.110348 (2024). 38823583 10.1016/j.radonc.2024.110348

[34] Almatroodi, S. A. et al. Potential therapeutic targets of Quercetin, a plant flavonol, and its role in the therapy of various types of cancer through the modulation of various cell signaling pathways. *Molecules*10.3390/molecules26051315 (2021). 33804548 10.3390/molecules26051315 PMC7957552

[35] Kimple, R. J. et al. Enhanced radiation sensitivity in HPV-positive head and neck cancer. *Cancer Res.* **73** (15), 4791–4800. 10.1158/0008-5472.CAN-13-0587 (2013). 23749640 10.1158/0008-5472.CAN-13-0587 PMC3732540

[36] Todorovic, V. et al. Mechanisms of different response to ionizing irradiation in isogenic head and neck cancer cell lines. *Radiat. Oncol.* **14**(1), 214. 10.1186/s13014-019-1418-6 (2019). 31775835 10.1186/s13014-019-1418-6 PMC6882348

[37] Arenz, A. et al. Increased radiosensitivity of HPV-positive head and neck cancer cell lines due to cell cycle dysregulation and induction of apoptosis. *Strahlenther. Onkol.* **190**(9), 839–46. 10.1007/s00066-014-0605-5 (2014). 24715240 10.1007/s00066-014-0605-5

[38] Gottgens, E. L. et al. Inhibition of CDK4/CDK6 enhances radiosensitivity of HPV negative head and neck squamous cell carcinomas. *Int. J. Radiat. Oncol. Biol. Phys.* **105**(3), 548–58. 10.1016/j.ijrobp.2019.06.2531 (2019). 31271827 10.1016/j.ijrobp.2019.06.2531

[39] Jansen, J. et al. A novel analysis method for evaluating the interplay of oxygen and ionizing radiation at the gene level. *Front. Genet.* **12**, 597635. 10.3389/fgene.2021.597635 (2021). 33995470 10.3389/fgene.2021.597635 PMC8113813

[40] Ha, J. et al. AZD7648, a DNA-PKcs inhibitor, overcomes radioresistance in Hep3B xenografts and cells under tumor hypoxia. *Am. J. Cancer Res.* **13** (10), 4918–4930 (2023). 37970336 PMC10636658

[41] Moon, S. U. et al. The expression of human papillomavirus type 16 (HPV16 E7) induces cell cycle arrest and apoptosis in radiation and hypoxia resistant glioblastoma cells. *Mol. Med. Rep.* **4**(6), 1247–53. 10.3892/mmr.2011.561 (2011). 21850378 10.3892/mmr.2011.561

[42] Ji, Q. et al. PYGL-mediated glucose metabolism reprogramming promotes EMT phenotype and metastasis of pancreatic cancer. *Int. J. Biol. Sci.* **19**(6), 1894–909. 10.7150/ijbs.76756 (2023). 37063425 10.7150/ijbs.76756 PMC10092766

[43] Lee, S. H. et al. p62/SQSTM1-induced caspase-8 aggresomes are essential for ionizing radiation-mediated apoptosis. *Cell Death Dis.* **12**(11), 997. 10.1038/s41419-021-04301-7 (2021). 34697296 10.1038/s41419-021-04301-7 PMC8546074

[44] Sorensen, B. S. et al. Effect of radiation on cell proliferation and tumor hypoxia in HPV-positive head and neck cancer in vivo models. *Anticancer Res.* **34** (11), 6297–6304 (2014). 25368228

[45] Gottgens, E. L., Bussink, J., Ansems, M., Hammond, E. M. & Span, P. N. AKT inhibition as a strategy for targeting hypoxic HPV-positive HNSCC. *Radiother. Oncol.* **149**, 1–7. 10.1016/j.radonc.2020.04.048 (2020). 32361013 10.1016/j.radonc.2020.04.048

[46] Le, Q. T. et al. Prognostic and predictive significance of plasma HGF and IL-8 in a phase III trial of chemoradiation with or without tirapazamine in locoregionally advanced head and neck cancer. *Clin. Cancer Res.* **18**(6), 1798–807. 10.1158/1078-0432.CCR-11-2094 (2012). 22383739 10.1158/1078-0432.CCR-11-2094 PMC3306471

[47] Gehrke, T. et al. Combination of salinomycin and radiation effectively eliminates head and neck squamous cell carcinoma cells in vitro. *Oncol. Rep.* **39**(4), 1991–8. 10.3892/or.2018.6267 (2018). 29436675 10.3892/or.2018.6267

[48] Meidenbauer, J. et al. Inhibition of ATM or ATR in combination with hypo-fractionated radiotherapy leads to a different immunophenotype on transcript and protein level in HNSCC. *Front. Oncol.* **14**, 1460150. 10.3389/fonc.2024.1460150 (2024). 39411143 10.3389/fonc.2024.1460150 PMC11473424

[49] Byers, L. A. et al. Serum signature of hypoxia-regulated factors is associated with progression after induction therapy in head and neck squamous cell cancer. *Mol. Cancer Ther.* **9**(6), 1755–63. 10.1158/1535-7163.MCT-09-1047 (2010). 20530716 10.1158/1535-7163.MCT-09-1047 PMC2913168

[50] Kondoh, N. & Mizuno-Kamiya, M. The role of immune modulatory cytokines in the tumor microenvironments of head and neck squamous cell carcinomas. *Cancers (Basel)*10.3390/cancers14122884 (2022). 35740551 10.3390/cancers14122884 PMC9221278

[51] Kindt, N. et al. Macrophage migration inhibitory factor in head and neck squamous cell carcinoma: Clinical and experimental studies. *J. Cancer Res. Clin. Oncol.* **139**(5), 727–37. 10.1007/s00432-013-1375-7 (2013). 23354841 10.1007/s00432-013-1375-7 PMC11824777

[52] Liu, L. et al. Blocking the MIF-CD74 axis augments radiotherapy efficacy for brain metastasis in NSCLC via synergistically promoting microglia M1 polarization. *J. Exp. Clin. Cancer Res.* **43**(1), 128. 10.1186/s13046-024-03024-9 (2024). 38685050 10.1186/s13046-024-03024-9 PMC11059744

[53] Gupta, Y., Pasupuleti, V., Du, W. & Welford, S. M. Macrophage migration inhibitory factor secretion is induced by ionizing radiation and oxidative stress in cancer cells. *PLoS One* **11**(1), e0146482. 10.1371/journal.pone.0146482 (2016). 26741693 10.1371/journal.pone.0146482 PMC4704778

[54] Dumitru, C. A. et al. Tumor-derived macrophage migration inhibitory factor modulates the biology of head and neck cancer cells via neutrophil activation. *Int. J. Cancer.* **129**(4), 859–69. 10.1002/ijc.25991 (2011). 21328346 10.1002/ijc.25991

[55] Pavon, M. A. et al. Enhanced cell migration and apoptosis resistance may underlie the association between high SERPINE1 expression and poor outcome in head and neck carcinoma patients. *Oncotarget* **6**(30), 29016–33. 10.18632/oncotarget.5032 (2015). 26359694 10.18632/oncotarget.5032 PMC4745708

[56] Schilling, D. et al. Induction of plasminogen activator inhibitor type-1 (PAI-1) by hypoxia and irradiation in human head and neck carcinoma cell lines. *BMC Cancer* **7**, 143. 10.1186/1471-2407-7-143 (2007). 17663760 10.1186/1471-2407-7-143 PMC1973081

[57] Artman, T. et al. Irradiation-induced regulation of plasminogen activator inhibitor type-1 and vascular endothelial growth factor in six human squamous cell carcinoma lines of the head and neck. *Int. J. Radiat. Oncol. Biol. Phys.* **76**(2), 574–82. 10.1016/j.ijrobp.2009.08.035 (2010). 20117293 10.1016/j.ijrobp.2009.08.035

[58] Bayer, C. et al. PAI-1 levels predict response to fractionated irradiation in 10 human squamous cell carcinoma lines of the head and neck. *Radiother. Oncol.* **86**(3), 361–8. 10.1016/j.radonc.2007.11.011 (2008). 18077030 10.1016/j.radonc.2007.11.011

[59] Zhu, C. et al. Prognostic significance and therapeutic potential of SERPINE1 in head and neck squamous cell carcinoma. *Cancer Med.* **14**(2), e70605. 10.1002/cam4.70605 (2025). 39817507 10.1002/cam4.70605 PMC11736624

[60] Koong, A. C. et al. Candidate genes for the hypoxic tumor phenotype. *Cancer Res.* **60** (4), 883–887 (2000). 10706099

[61] O’Rorke, M. A. et al. Human Papillomavirus related head and neck cancer survival: A systematic review and meta-analysis. *Oral Oncol.* **48**(12), 1191–201. 10.1016/j.oraloncology.2012.06.019 (2012). 22841677 10.1016/j.oraloncology.2012.06.019

[62] Zhou, C. & Parsons, J. L. The radiobiology of HPV-positive and HPV-negative head and neck squamous cell carcinoma. *Expert Rev. Mol. Med.* **22**, e3. 10.1017/erm.2020.4 (2020). 32611474 10.1017/erm.2020.4 PMC7754878

[63] Liu, Q. et al. Subjugation of TGFβ signaling by Human Papilloma Virus in head and neck squamous cell carcinoma shifts DNA repair from homologous recombination to alternative end joining. *Clin. Cancer Res.* **24**(23), 6001–14. 10.1158/1078-0432.CCR-18-1346 (2018). 30087144 10.1158/1078-0432.CCR-18-1346

[64] Liu, T. et al. CENPM upregulation by E5 oncoprotein of Human Papillomavirus promotes radiosensitivity in head and neck squamous cell carcinoma. *Oral Oncol.* **129**, 105858. 10.1016/j.oraloncology.2022.105858 (2022). 35462155 10.1016/j.oraloncology.2022.105858

[65] James, C. D. et al. HPV16 genome structure analysis in oropharyngeal cancer PDXs identifies tumors with integrated and episomal genomes. *Tumour Virus Res.* **18**, 200285. 10.1016/j.tvr.2024.200285 (2024). 38936774 10.1016/j.tvr.2024.200285 PMC11261002

[66] Deneka, A. Y. et al. Association of TP53 and CDKN2A mutation profile with tumor mutation burden in head and neck cancer. *Clin. Cancer Res.* **28**(9), 1925–37. 10.1158/1078-0432.CCR-21-4316 (2022). 35491653 10.1158/1078-0432.CCR-21-4316 PMC9186806

[67] Li, Y. et al. HPV-driven rewiring of the tumor immune microenvironment through single-cell profiling informs prognosis and therapy in HNSCC. *Oral Oncol.* **172**, 107789. 10.1016/j.oraloncology.2025.107789 (2026). 41314067 10.1016/j.oraloncology.2025.107789

