# Supplementary material for: Hypoxia, metastatic origin and HPV16 E6/E7 expression differentially shape the radiation response in head and neck squamous cell carcinoma cell lines
Source: Sci Rep. 2026 May 22;16:23306. doi: 10.1038/s41598-026-54319-0 (PMC13402748; doi:10.1038/s41598-026-54319-0)
Supplement: Supplementary file 2 — Supplementary Material 2 [file 41598_2026_54319_MOESM4_ESM.pdf]

Hypoxia, metastatic origin and HPV16 E6/E7 expression differentially shape the radiation response in head and neck squamous cell carcinoma cell lines

Jana Pereckova[1], Filip Zavadil Kokas[2], Simona Voznicova[3], Ondrej Vasicek[3], Jitka Holcakova[2], Roman Hrstka[2]*, Tomas Perecko[1]*

[1] Department of Cell Biology and Radiobiology, Institute of Biophysics of the Czech Academy of Sciences, Kralovopolska 135, 612 00 Brno, Czech Republic
[2] Research Centre for Applied Molecular Oncology, Masaryk Memorial Cancer Institute, Zluty kopec 7, Brno, 656 53, Czech Republic
[3] Department of Biophysics of Immune System, Institute of Biophysics of the Czech Academy of Sciences, Kralovopolska 135, 612 00 Brno, Czech Republic

- Authors to whom correspondence should be addressed: roman.hrstka@mou.cz, tomas.perecko@ibp.cz

Supplementary Information Files:
Figure S1
Figure S2
Table ST1
Figure S3
Figure S4

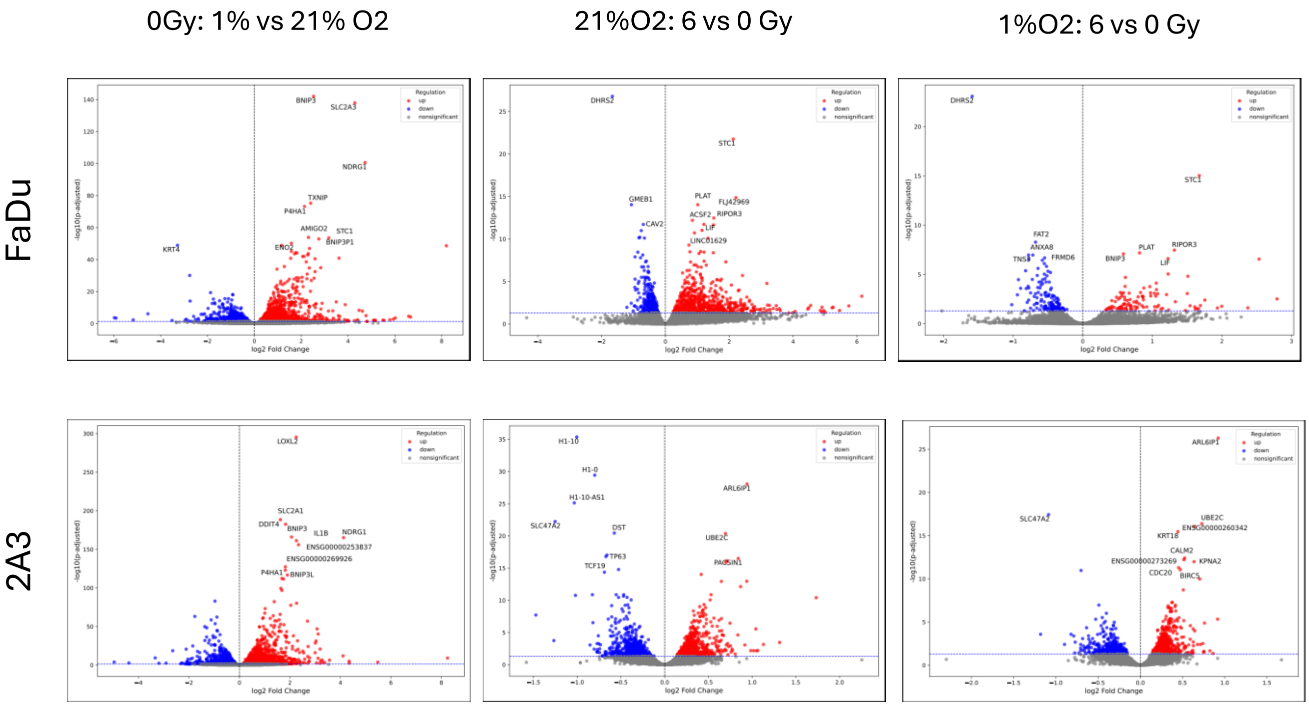

Figure S1. Effects of hypoxia and gamma-irradiation on differentially expressed genes in FaDu and 2A3 cell lines. Data are presented as log2 fold change versus negative decimal logarithm of the adjusted p-value. The ten most significantly altered genes are annotated. Threshold applied: adjusted p-value < 0.1.

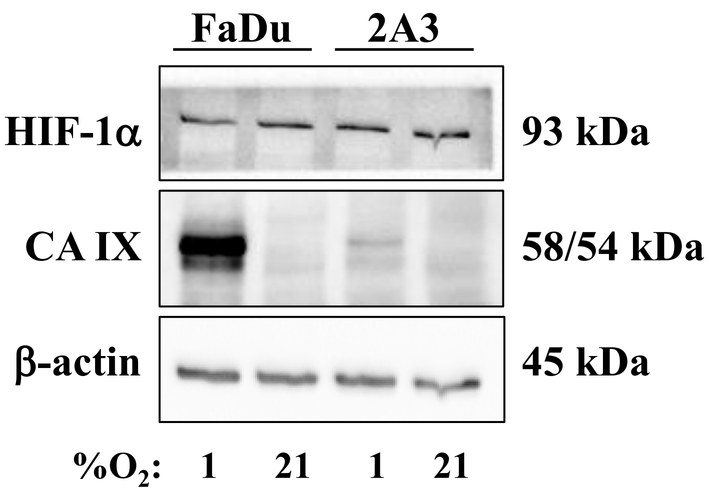

Figure S2. Representative proof-of-concept protein visualization of HIF1α and CA IX in FaDu and 2A3 cell lines cultured under 1 or 21% oxygen. Representative figure, n=1, beta-actin was used as loading control.

symbol	FADU_hypoxia_log2FoldChange	FADU_hypoxia_padj	2A3_hypoxia_log2FoldChange	2A3_hypoxia_padj
ADM	0.860004978	0.004212433	0.931317778	6.54E-07
AK4	1.001251976	1.09E-11	0.875760183	6.98E-34
ANGPTL4	1.271765031	0.073856256	2.381152849	4.44E-17
ANKRD37	1.044074298	2.75E-05	0.812694224	0.003597834
BNIP3	2.527482131	8.44E-143	2.063469956	9.94E-167
BNIP3L	1.660519877	1.17E-22	1.900286565	1.82E-117
CA9	8.20136887	2.36E-49	8.244657919	7.22E-10
DDIT4	2.28151417	1.25E-43	1.827979588	3.07E-183
EGLN3	1.279762247	2.19E-36	1.640059455	3.90E-100
HK2	1.770241669	5.11E-45	1.01863884	6.40E-74
LDHA	1.198589811	1.29E-20	0.923608429	2.99E-61
LOXL2	2.081375677	8.83E-24	2.249182335	3.74E-296
NDRG1	4.727211924	2.52E-101	4.12470853	6.41E-166
P4HA1	2.142657467	6.08E-74	1.815073555	1.12E-123
PDK1	2.063000403	1.15E-42	1.653531166	2.77E-44
PFKFB3	1.151258587	1.23E-17	1.084056448	2.15E-46
PGK1	0.876632446	6.34E-07	0.953616801	4.14E-51
PLOD2	1.31036022	6.29E-15	1.682053323	5.19E-113
SERPINE1 ENSG00000106366	2.244723434	9.08E-29	2.033785899	2.94E-78
SLC2A1	1.822798177	1.89E-27	1.620835252	3.24E-189
SLC2A3	4.291676279	1.18E-138	3.19513168	1.58E-21

Table ST1. Differential gene expression analysis of FaDu and 2A3 head and neck squamous cell carcinoma (HNSCC) cell lines cultured under hypoxic (1% O_2) compared with normoxic (21% O_2) conditions. The analysis identifies significant upregulation of established HIF-1 target genes involved in metabolic adaptation (SLC2A1/3, HK2, PDK1, LDHA), survival and autophagy (BNIP3, BNIP3L, DDIT4), extracellular matrix remodeling (P4HA1, PLOD2, LOXL2), and angiogenic signaling (ADM, ANGPTL4). Log_2 fold changes and adjusted p values are shown for each gene and cell line.

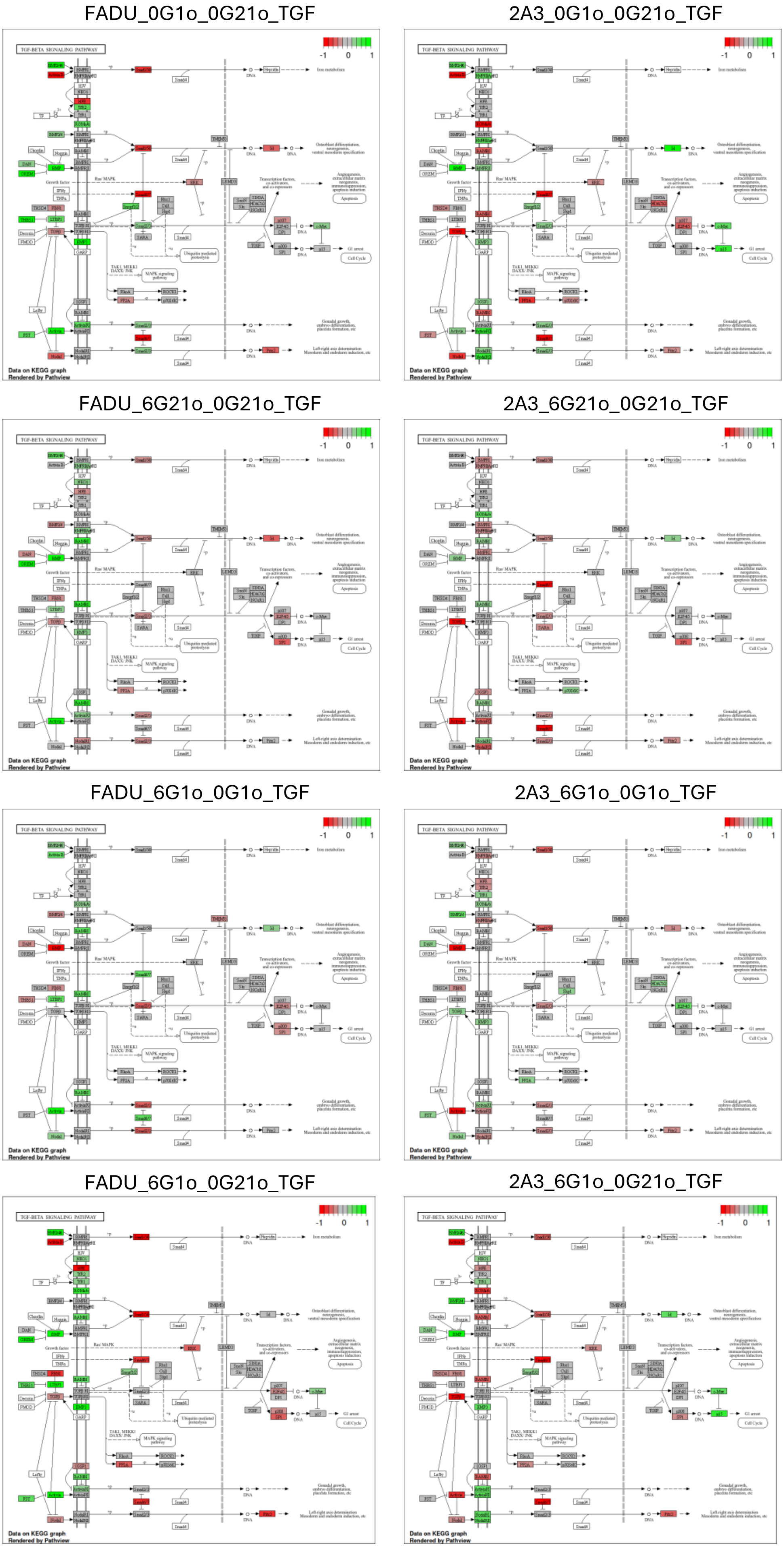

Figure S3. KEGG TGF-beta signaling pathway with mapped differentially expressed genes from FaDu and 2A3 cell lines after hypoxia and gamma-irradiation. Genes are color-coded according to log2 fold change: red indicates downregulation, green indicates upregulation, and grey denotes no significant change. Threshold applied: adjusted p-value < 0.1. The map was generated using the KEGG database (DOI: 10.1093/nar/28.1.27) and visualized with iDep (DOI: 10.1186/s12859-018-2486-6).

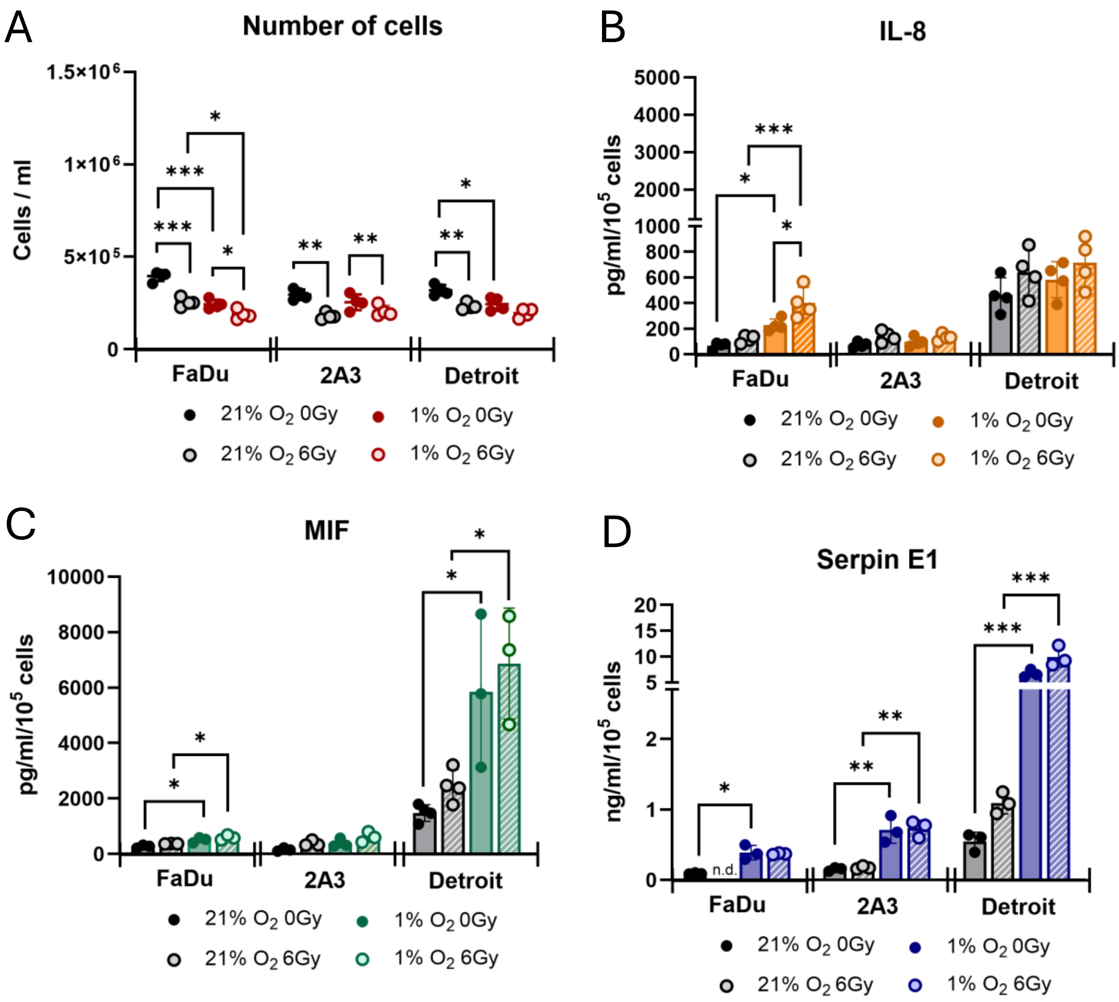

Figure S4. Effects of hypoxia and gamma-irradiation on cytokine/chemokine release in HNSCC 24 hours after gamma irradiation. (A) Cell numbers of FaDu, 2A3, and Detroit-562 cultured under normoxic (21% O_2) or hypoxic (1% O_2) conditions, assessed 24 hours after 6 Gy gamma-irradiation. Data represent mean ± SD from four independent experiments (n = 4). Statistical analysis: two-way ANOVA with Tukey's multiple comparisons test; *p < 0.05, **p < 0.01, ***p < 0.001. Levels of IL-8 (B), MIF (C), and Serpin E1 (D) in supernatants from FaDu, 2A3, and Detroit-562 cells cultured under normoxic or hypoxic conditions, assessed 24 hours after gamma-irradiation. Concentrations were normalized to cell numbers per condition. Data represent mean ± SD from three to four independent experiments (n = 3–4). Statistical analysis: two-way ANOVA with Tukey's multiple comparisons test; *p < 0.05, **p < 0.01, ***p < 0.001.
