# Supplementary material for: Hypoxia, metastatic origin and HPV16 E6/E7 expression differentially shape the radiation response in head and neck squamous cell carcinoma cell lines
Source: Sci Rep. 2026 May 22;16:23306. doi: 10.1038/s41598-026-54319-0 (PMC13402748; doi:10.1038/s41598-026-54319-0)
Supplement: Supplementary file 4 — Supplementary Material 4 [file 41598_2026_54319_MOESM3_ESM.pdf]

Hypoxia, metastatic origin and HPV16 E6/E7 expression differentially shape the radiation response in head and neck squamous cell carcinoma cell lines

Jana Pereckova[1], Filip Zavadil Kokas[2], Simona Voznicova[3], Ondrej Vasicek[3], Jitka Holcakova[2], Roman Hrstka[2]*, Tomas Perecko[1]*

[1] Department of Cell Biology and Radiobiology, Institute of Biophysics of the Czech Academy of Sciences, Kralovopolska 135, 612 00 Brno, Czech Republic
[2] Research Centre for Applied Molecular Oncology, Masaryk Memorial Cancer Institute, Zluty kopec 7, Brno, 656 53, Czech Republic
[3] Department of Biophysics of Immune System, Institute of Biophysics of the Czech Academy of Sciences, Kralovopolska 135, 612 00 Brno, Czech Republic

- Authors to whom correspondence should be addressed: roman.hrstka@mou.cz, tomas.perecko@ibp.cz

1 – 21% oxygen / 0 Gy
2 – 21% oxygen / 6 Gy
3 – 1% oxygen / 0 Gy
4 – 1% oxygen / 6 Gy

2024-05-27 WB FaDu

cleaved caspase-3 17,19 kDa

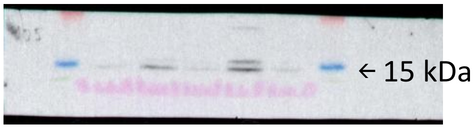

← 15 kDa

beta-actin 42 kDa

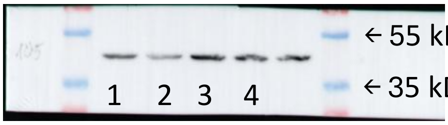

← 55 kDa
← 35 kDa

2024-06-03 WB FaDu

cleaved caspase-3 17,19 kDa

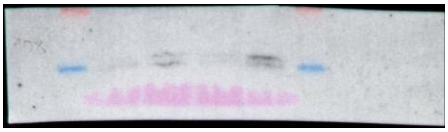

beta-actin 42 kDa

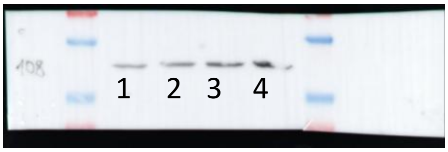

2025-03-06 WB FaDu

cleaved caspase-3 17,19 kDa

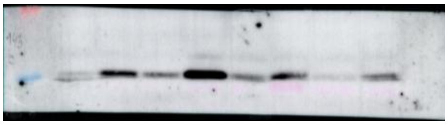

2025-03-06 WB FaDu – different filter

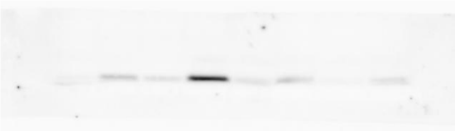

beta-actin 42 kDa

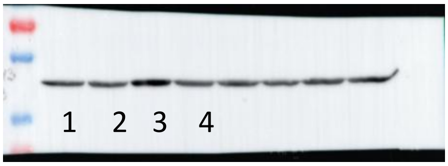

2025-01-20 WB 2A3 | 2025-01-27 WB 2A3

cleaved caspase-3 17,19 kDa

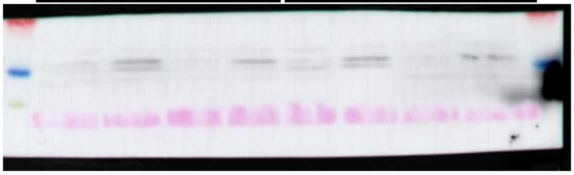

beta-actin 42 kDa

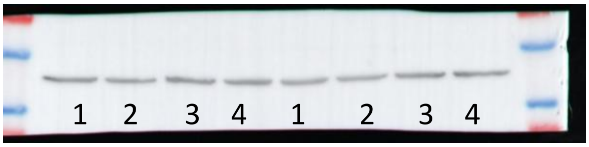

2025-02-03 WB 2A3

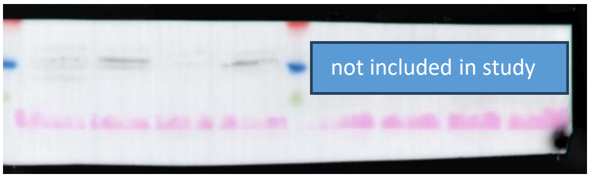

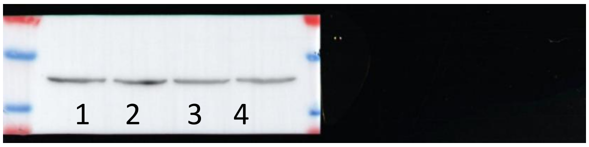

2025-05-12 WB Detroit | 2025-05-19 WB Detroit | 2025-05-26 WB Detroit

cleaved caspase-3 17,19 kDa

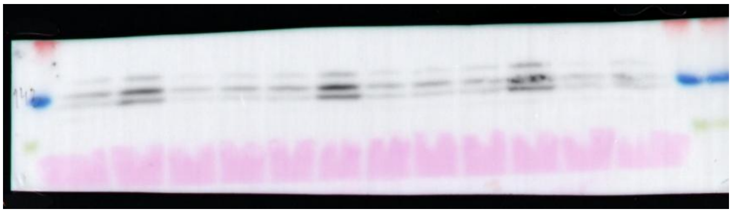

beta-actin 42 kDa

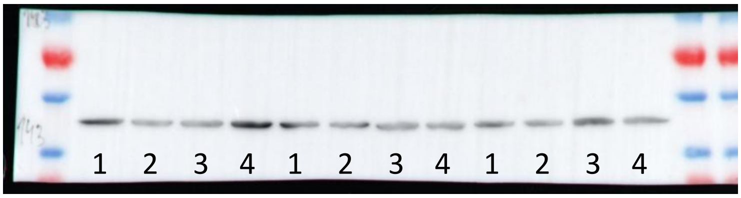

1 – 21% oxygen / 0 Gy
2 – 21% oxygen / 6 Gy
3 – 1% oxygen / 0 Gy
4 – 1% oxygen / 6 Gy

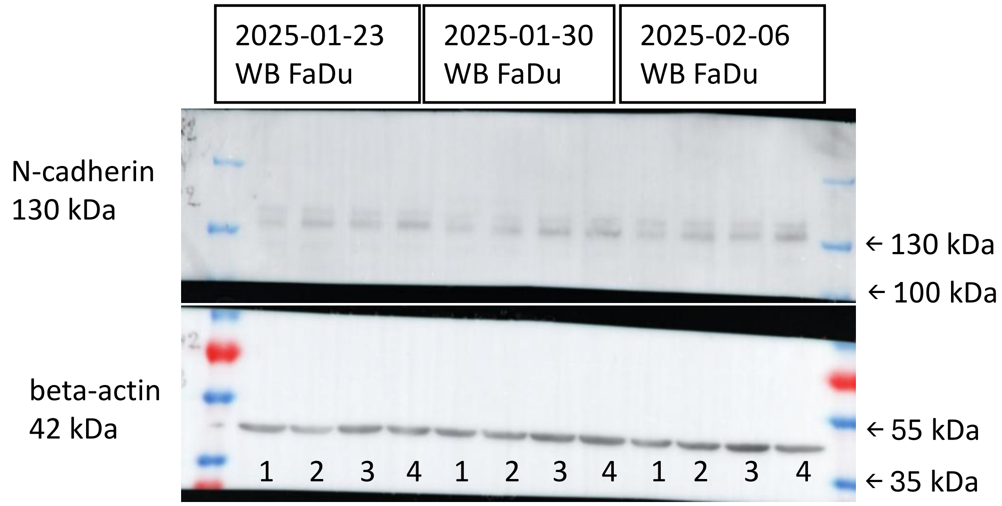

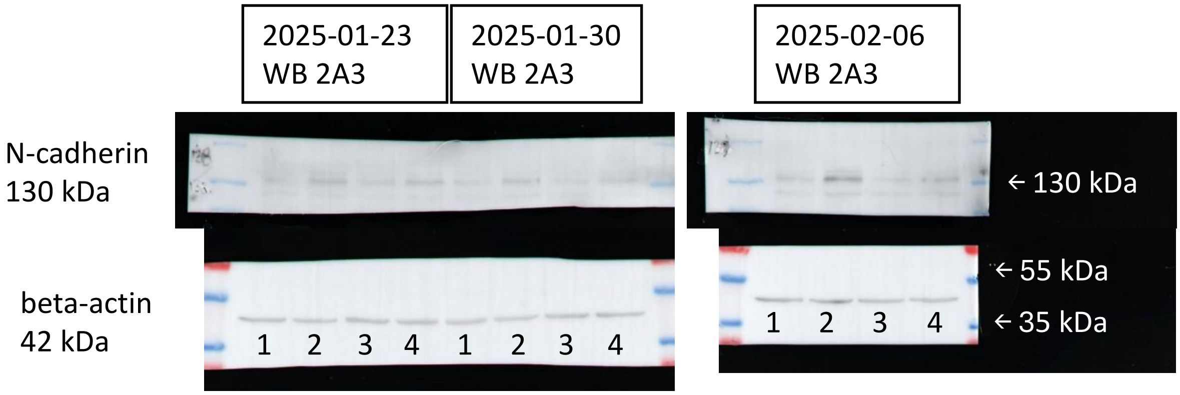

1 – 21% oxygen / 0 Gy
2 – 21% oxygen / 6 Gy
3 – 1% oxygen / 0 Gy
4 – 1% oxygen / 6 Gy

2025-01-23 WB FaDu	2025-01-30 WB FaDu

E-cadherin
125 kDa

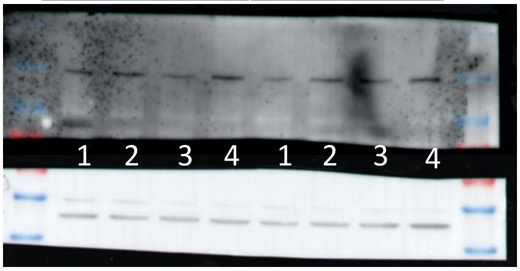

← 130 kDa
← 100 kDa

beta-actin
42 kDa

← 55 kDa
← 35 kDa

2025-02-06
WB FaDu

E-cadherin
125 kDa

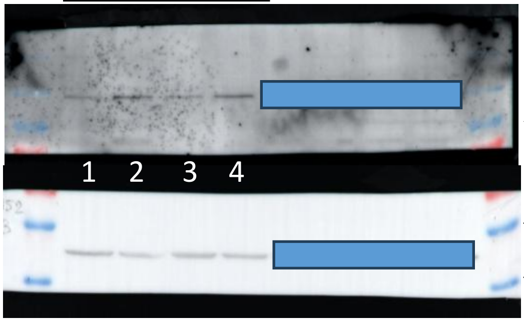

← 130 kDa
← 100 kDa

beta-actin
42 kDa

← 55 kDa
← 35 kDa

2025-01-23 WB 2A3	2025-01-30 WB 2A3

E-cadherin
125 kDa

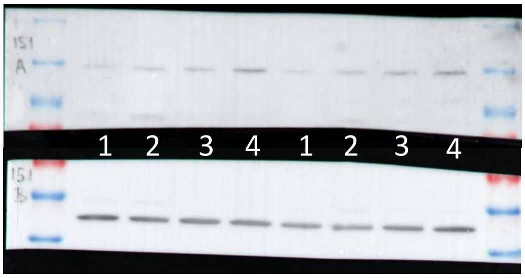

← 130 kDa
← 100 kDa

beta-actin
42 kDa

← 55 kDa
← 35 kDa

2025-02-06
WB 2A3

E-cadherin
125 kDa

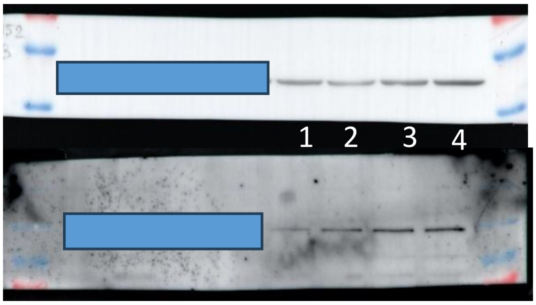

← 130 kDa
← 100 kDa

beta-actin
42 kDa

← 55 kDa
← 35 kDa

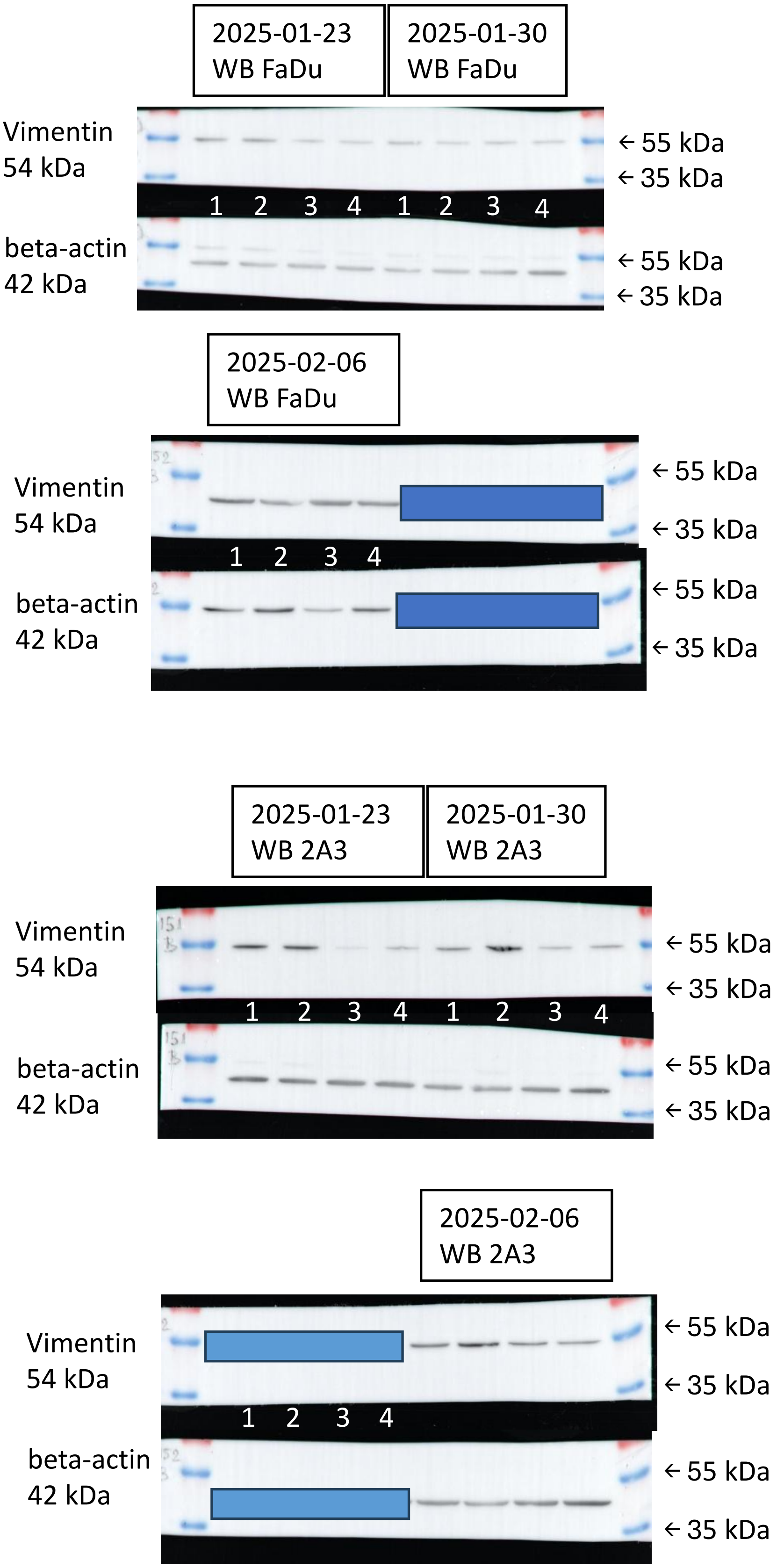

1 – 21% oxygen / 0 Gy
2 – 21% oxygen / 6 Gy
3 – 1% oxygen / 0 Gy
4 – 1% oxygen / 6 Gy

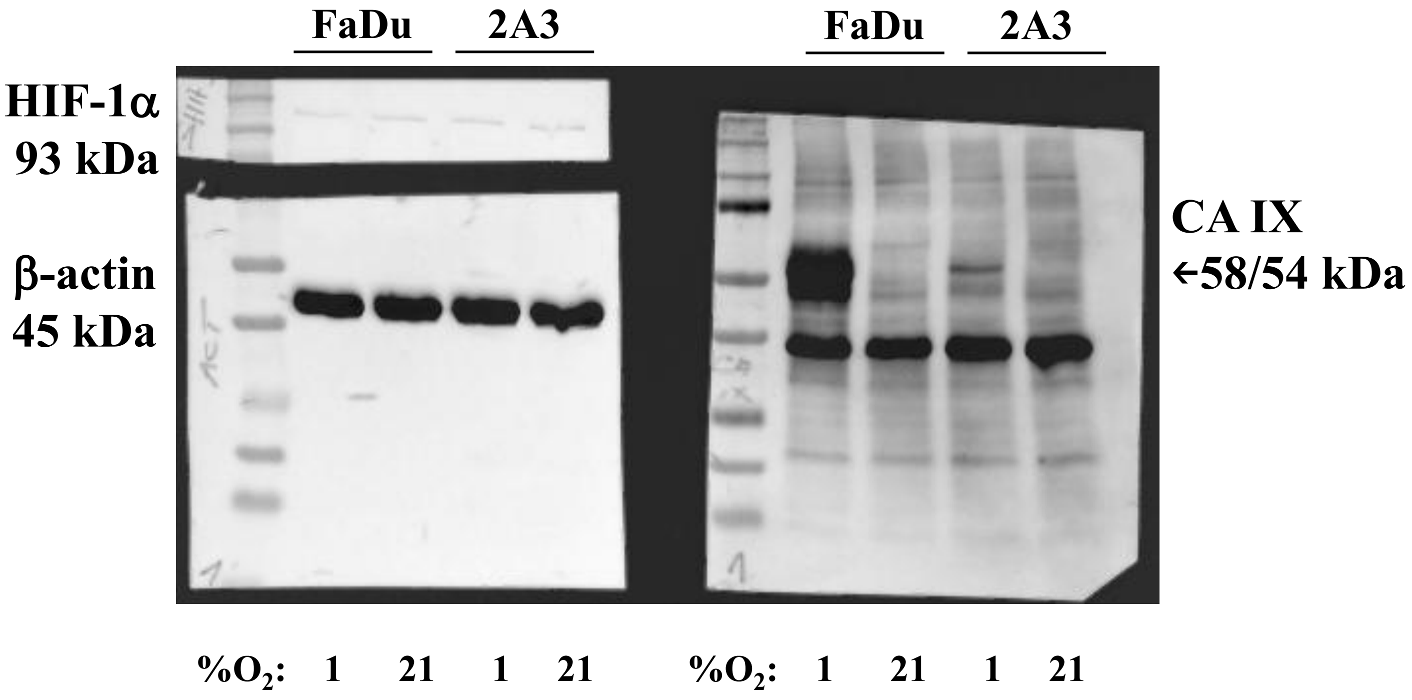
FaDu
2A3
FaDu
2A3
HIF-1α
93 kDa
β-actin
45 kDa
CA IX
←58/54 kDa
%O2: 1 21 1 21
%O2: 1 21 1 21
