# Supplementary material for: Hypoxia, metastatic origin and HPV16 E6/E7 expression differentially shape the radiation response in head and neck squamous cell carcinoma cell lines
Source: Sci Rep. 2026 May 22;16:23306. doi: 10.1038/s41598-026-54319-0 (PMC13402748; doi:10.1038/s41598-026-54319-0)
Supplement: Supplementary file 5 — Supplementary Material 5 [file 41598_2026_54319_MOESM5_ESM.docx]

**Supplementary information**

Supplementary file 1: DEGs_genes_FADU_and_2A3 (XLSX format)

Contains differentially expressed genes analyses used in this study.

Supplementary file 2: SI-Figures (PDF format)

Contains Figures S1–S4 showing transcriptomic and pathway analyses of HNSCC cell lines under hypoxia and gamma-irradiation, including volcano plots, HIF1α and CA IX protein levels, KEGG pathway maps (TGFβ), and ELISA data on cytokine/chemokine release 24 hours after irradiation and Table ST1 with differential gene expression analysis of FaDu and 2A3 cultured under hypoxic (1% O₂) and normoxic (21% O₂) conditions.

Supplementary file 3: TCGA_analysis_genes (XLSX format)

Contains analyses of differentially expressed genes for the comparison of HPV‑positive versus HPV‑negative gene signatures (HPVpos_vs_HPVneg) and genes associated with metastatic behavior (Npos_vs_N0).

Supplementary file 4: Original western blot membranes (PDF format)

Contains original western blot membranes used in this study, grouped by target protein. Membranes are labelled to correspond to the relevant main‑text figures and panels.
